# Multiple Dimensions Define Thresholds for Population Resilience of the Eastern Oyster, *Crassostrea virginica*

**DOI:** 10.1002/ece3.70759

**Published:** 2025-01-21

**Authors:** Megan K. La Peyre, Hongqing Wang, Shaye E. Sable, Wei Wu, Bin Li, Devin Comba, Carlos Perez, Melanie Bates, Lauren M. Swam

**Affiliations:** ^1^ U.S. Geological Survey Louisiana Cooperative Fish and Wildlife Research Unit Baton Rouge Louisiana USA; ^2^ School of Renewable Natural Resources Louisiana State University Agricultural Center Baton Rouge Louisiana USA; ^3^ U.S. Geological Survey Wetland and Aquatic Research Center Baton Rouge Louisiana USA; ^4^ Dynamic Solutions LLC Baton Rouge Louisiana USA; ^5^ Division of Coastal Sciences, School of Ocean Science and Engineering The University of Southern Mississippi Ocean Springs Mississippi USA; ^6^ Department of Experimental Statistics Louisiana State University Baton Rouge Louisiana USA; ^7^ GEC Inc Baton Rouge Louisiana USA

**Keywords:** *Crassostrea virginica*, ecological threshold, estuary, Gulf of Mexico, salinity

## Abstract

A species' distribution depends on its tolerance to environmental conditions. These conditions are defined by a minimum, maximum, and optimal ranges of single and combined factors. Forays into environmental conditions outside the minimum or maximum tolerance of a species (i.e., thresholds) are predicted to have large effects on a species' population and may help predict population resilience in the face of changing conditions. Here, we explore ecological thresholds for an important fisheries species and ecosystem engineer, *Crassostrea virginica* (eastern oyster). In coastal Louisiana, extreme freshwater inputs from rivers and precipitation events impact estuarine salinity, which is a key driver of oyster population dynamics. Using daily salinity and monthly oyster abundance monitoring data across Louisiana estuaries, we explore low salinity exposure threshold levels for oysters. Two statistical approaches were applied, with each model highlighting a different operational definition of a threshold: random forest models identified a threshold as an abrupt change in the oyster abundance‐ salinity relationship, while Bayesian models identified an increased probability of oyster abundance dropping below a critical threshold, defined here as less than 50% of the 5‐year mean. All model results indicate oysters in coastal Louisiana experience low salinity exposure thresholds, defined as the number of consecutive summer days of salinity levels less than 5. However, actual number of days and salinity threshold differed by statistical approach, oyster life stage, and estuary highlighting the multiple dimensions defining ecological thresholds. While thresholds are considered important benchmarks to inform management and assess population or ecosystem vulnerability, our results reveal the need to carefully relate threshold definition to management goals and to acknowledge that thresholds may be highly context dependent.

## Graphical abstract

Thresholds or tipping points are generally defined as a point or a narrow transitional zone at which an organisms' abundance changes abruptly in response to relatively small changes in a driving force. Here, we use two statistical approaches to highlight different operational definitions of a threshold using the largely sessile marine invertebrate, *Crassostrea virginica* , and its response to low salinity in coastal Louisiana, USA as our test case. While thresholds are considered important benchmarks to inform management and assess population or ecosystem vulnerability, our results reveal the need to carefully relate threshold definition to management goals and to acknowledge that thresholds may be highly context dependent.

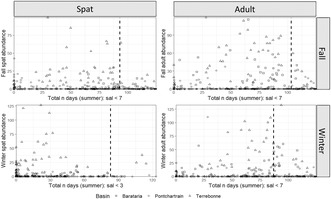

## 1 Introduction

A species' presence and population success in a location depend on its tolerance to local environmental conditions. These conditions are defined by minimum, maximum, and optimal ranges of single and combined factors (Shelford's Law of Tolerance; Shelford 1931). Knowing the limiting factors impacting survival and reproductive success critically informs management for species and helps predict population resilience in the face of changing conditions (Scheffer et al. 2001, 2009; Folke et al. 2004; Hiddink and Kaiser 2005; Tang and Riley 2021). Benthic and largely sessile marine invertebrates located within estuarine environments experience varying conditions from overlying water conditions influenced by both marine and terrestrial forces. Many of these organisms have evolved strategies to tolerate large ranges of conditions and an ability to adapt to constant changes (Bayne 2017; Gosling 2021). Still, their distribution and survival across these estuarine environments remain controlled by single and combined conditions that present optimal, stress, or intolerance zones, defined by thresholds, over different temporal and spatial scales. Thresholds or tipping points are generally defined as a point or a narrow transitional zone at which an ecosystem's structural or functional property changes abruptly in response to relatively small changes in a driving force (Scheffer et al. 2001; Folke et al. 2004; Larsen and Alp 2015; Toms and Villard 2015) but may also be defined as when driving forces cause this property to drop below a critical defined level (Ficetola and Denoël 2009). Identifying these thresholds for key species helps inform individual and ecosystem management and predict future changes, particularly in a time with predicted increases in extreme events (Jentsch, Kreyling, and Beierkuhnlein 2007; Carrier‐Belleau et al. 2023; Luo et al. 2024).

Threshold detection proves challenging for ecological systems (e.g., Dudney and Suding 2020). While the detection of thresholds remains a priority goal for species and ecosystem management, debate over our ability to detect thresholds in ecological datasets, the operational definition of a threshold, appropriate data exploration methods, and the identification of appropriate organisms or systems persists (i.e., Hiddink and Kaiser 2005; Ficetola and Denoël 2009; Hillebrand et al. 2020, 2023). Thresholds may be difficult to detect in situations where multiple factors may limit an organism (or indicator variable), or when other nonlinear dynamics may dominate responses (Hiddink and Kaiser 2005; Hillebrand et al. 2020). For individual species, thresholds below or above which species exist and reproduce successfully may be defined by multiple dimensions, and may shift as a function of numerous factors, including season, life‐history stage, prior or recent exposure, and additive or synergistic interactions which can mask threshold detection (Hiddink and Kaiser 2005; Cote, Darling, and Brown 2016; Truebano et al. 2018; Hillebrand et al. 2020; Carrier‐Belleau et al. 2023). In addition, thresholds can be defined by multiple dimensions, including magnitude, duration, and rate of change in a controlling parameter.

Studies to determine thresholds also often adopt different operational definitions of a critical threshold. For example, in some cases, abrupt changes in the relationship between controlling and response variables are used to define a critical threshold; in other cases, a critical threshold is defined by exploring when the probability of the response variable crossing a predetermined level occurs (e.g., 50% of the 5‐year mean; Ficetola and Denoël 2009). Numerous approaches have been used to explore one of these types of thresholds including generalized linear models, generalized additive models, and broken stick regressions. Recent studies have demonstrated that the use of random forest (RF) methods (i.e., Roubeix et al. 2016; Isabel et al. 2021; Rostami et al. 2021) and Bayesian analyses (i.e., Clark 2005; Wu et al. 2018) provide statistical means to detect potential thresholds. While addressing the same question, the different methods employ somewhat different operational definitions of thresholds: Bayesian approaches employ a probabilistic approach, identifying where the response variable (i.e., organism abundance) falls below a critical value with more than 50% probability, while RF approaches identify thresholds where abrupt changes in the relationship between controlling and the response variable may occur. The choice of approach and threshold definition may thus depend on the question of interest.

Here, we explore the use of field‐based abundance and environmental data of a valuable fishery species and ecosystem engineer, the eastern oyster, *Crassostrea virginica* , in the northern Gulf of Mexico (nGoM) to explore the threshold concept and identify potential thresholds limiting its distribution and production. Across its range, oysters serve as foundation species, provide valuable ecosystem services, and support a productive fishery (Coen and Humphries 2017). Maintenance of the integrity of reefs created by oysters depends on their recruitment and growth (Soniat et al. 2012), but also survival through periods of stress to ensure the habitat (reef) developed by the oyster may continue to exist and support other fisheries, benthic‐pelagic coupling, wave attenuation, and future oyster settlement (Coen and Humphries 2017; Morris et al. 2021; Swam et al. 2022). While the success of oyster restoration and aquaculture operations can be influenced to some extent by management, their outcomes are constrained by overlying water characteristics across oyster reef‐supporting and producing areas. As resource conditions (environmental factors) shift away from long‐term means and extremes become more common (e.g., Prein et al. 2017; Feng et al. 2023), the probability of situations exceeding critical thresholds can increase dramatically with more frequent forays beyond the critical thresholds of tolerance for species, which can dramatically alter the realized niche of individual species (Jentsch, Kreyling, and Beierkuhnlein 2007; Myhre et al. 2019; AghaKouchak et al. 2020; Luo et al. 2024). These thresholds may provide key points to anticipate dramatic changes in population distribution and to ensure the success of management and restoration efforts (Harley et al. 2017; Rilov et al. 2019). Zabin et al. (2022) recently identified a lack of planning for extreme and acute events as a contributing factor to failure of management, conservation, and restoration.

Existing studies on the eastern oyster highlight that this largely sessile estuarine species' growth, reproduction, survival, and distribution across its range are predominantly driven by salinity and temperature regimes (Shumway 1996). Due to these two dominant drivers, this species is an ideal candidate to explore critical thresholds related to its distribution and future survival in the face of changing climate. While other factors have been identified as controlling oyster survival or condition, including low dissolved oxygen events and acidification, salinity and its interaction with temperature tend to overwhelmingly predict population and physiological response of oysters (Rybovich et al. 2016; Southworth, Long, and Mann 2017; Casas et al. 2018; Marshall, Casas, et al. 2021; Marshall, Coxe, et al. 2021; Pruett et al. 2021; McFarland Vignier et al. 2022; Sirovy et al. 2023). Heatwaves and extreme precipitation events have increased in frequency, intensity, and duration across many regions of the globe with models predicting further intensification of these trends (Bindoff et al. 2019; Myhre et al. 2019; Luo et al. 2024). For example, across the United States, the frequency of extreme heat waves has increased 300% over the last 5 decades, while the length of the heat wave season has increased over threefold (NOAA 2022). In coastal waters along the nGoM where eastern oysters are a dominant reef‐building organism and an important fishery, similar trends and predictions exist. Specifically, water temperatures have warmed significantly during the fall to spring seasons and some estuarine areas have experienced high freshwater inflow from upstream precipitation, reducing average salinity in oyster‐growing estuaries with these trends expected to continue (Prein et al. 2017; Feng et al. 2023) increasing the likelihood of oyster's exposure to low‐salinity events, which have proven lethal (Gledhill et al. 2020; Du et al. 2021). Here, we explore fishery‐independent monitoring data on oysters across coastal Louisiana, in conjunction with daily salinity datasets to identify thresholds of tolerance for different life stages of the eastern oyster that incorporate both seasonal timing (i.e., temperature interactions), intensity (low salinity threshold), and exposure (total number of days, continuous number of days) of the stressor (salinity).

## 2 Methods

Using continuous data recorder daily output, and satellite‐derived data we created interpolated coast‐wide salinity layers following methods detailed in Swam et al. (2022). We acquired Louisiana Department of Wildlife and Fisheries fishery independent oyster monitoring data, collected by 24″ dredge which provides oyster size and abundance at approximately 80 fixed stations across Louisiana (LDWF 2018a). We examined the oyster abundance and density data by size class (< 25 mm shell height SH = spat; > 75 mm SH = adult) in relation to counts of total days and maximum continuous days of exposure to salinity below identified thresholds (intensity) of salinity levels of 1, 3, 5, 7, and 9 through a calendar year, and during hot summer months (May 1–Aug 31) for the years 2016–2020 as detailed below. We applied RF, a machine learning algorithm, and multilevel models in Bayesian inference, to derive salinity value and exposure thresholds to oyster abundance.

### 2.1 Study Area

**FIGURE 1:**
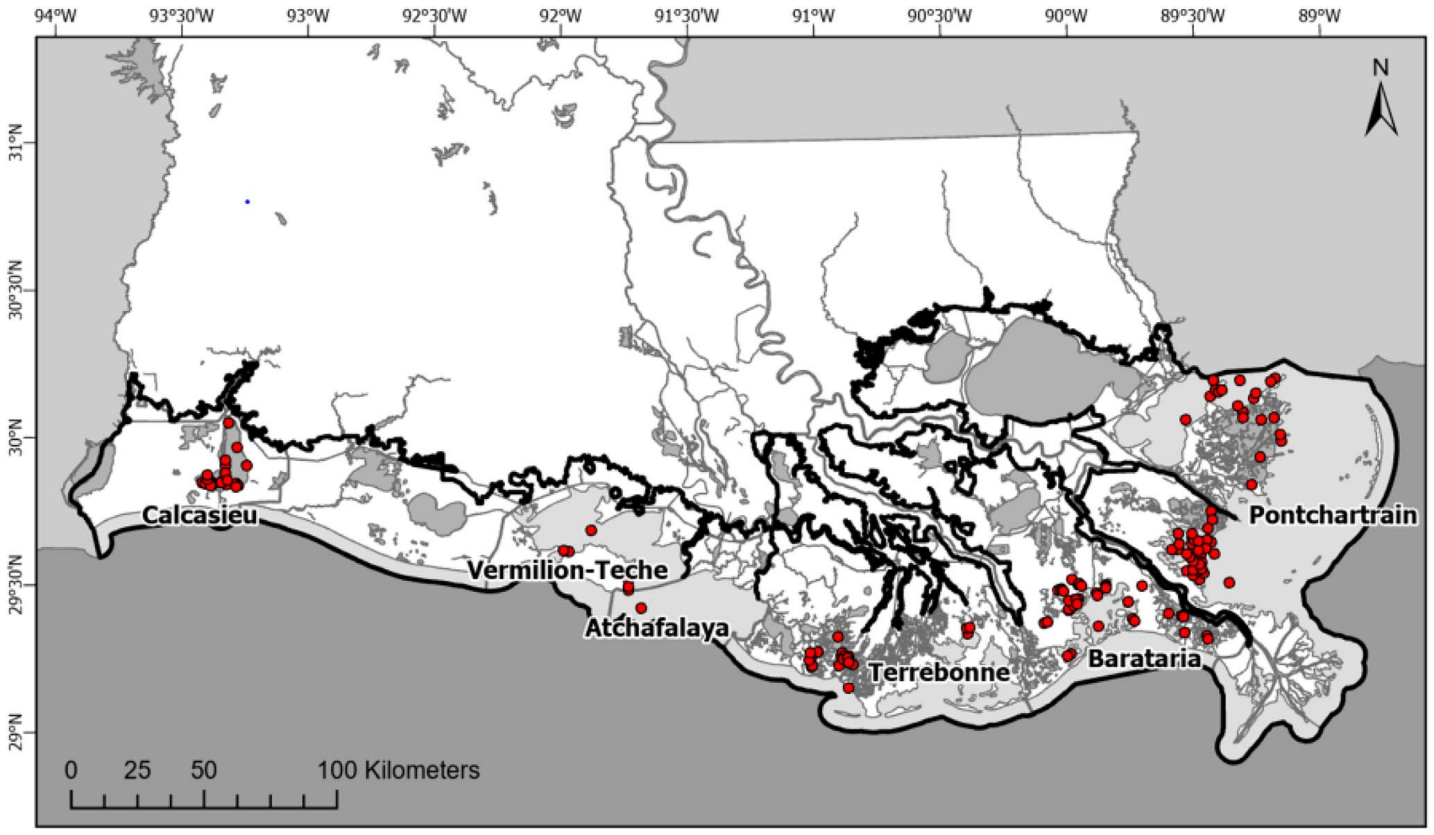
Study area map. Coastal Louisiana study basins (outlined in gray and labeled), and long‐term oyster sampling stations (LDWF 2018) sampled by the Louisiana Department of Wildlife and Fisheries. The black line indicates the study area boundary used to develop salinity interpolations (Swam et al. 2022). The map background is from the National Land Cover Dataset (NLCD 2016).

Louisiana estuarine basin boundaries extended 5 km from the coastline were used to define the study area, encompassing 100% of Louisiana's public seed grounds and private oyster lease bottoms (Figure 1). Across coastal Louisiana, oysters are ubiquitous and found within every major basin and estuary (LDWF 2020). Across all the basins, approximately 680,000 ha are managed as public oyster grounds and 165,000 ha are managed as private leases for oyster production (LDWF 2018a). While only a subset of existing oyster reefs and managed areas are mapped (i.e., < 25,000 ha; LDWF 2018a; La Peyre, Marshall, and Sable 2021), a recent study in coastal Louisiana using water quality indicators to map oyster resource zones identified close to 1.8 M ha of estuarine and coastal area potentially supportive of oyster survival and production (Swam et al. 2022). For this work, we used data for east Louisiana basins (Pontchartrain, Barataria, Terrebonne) and west Louisiana basins (Atchafalaya, Vermilion‐Teche, Calcasieu). Two Louisiana management basins were not included in analyses: Sabine Basin was not included in analyses due to poor resolution on daily salinity data (Swam et al. 2022), and Outside Waters were not included due to this study area crossing multiple estuarine basins.

### 2.2 Oyster Abundance Data

We accessed publicly available data collected through the Louisiana Department of Wildlife and Fisheries Fishery Independent Monitoring Program for oysters (LDWF 2018a; FisheriesDataDownload (la.gov), “Oyster Biological”). This monitoring program collects approximately monthly dredge (24″) oyster size class and abundance data from 101 stations coast wide. The 2016–2020 data were downloaded, providing oyster abundance by 5 mm increment shell height size classes. Data were collated by size distribution into counts of “spat” (shell height < 25 mm), and “adult” (shell height ≥ 25 mm) for each sample date. We analyzed fall (September and October) dredge abundances (spat, adult), winter (November and December) dredge abundances (spat, adult), and square meter density (spat, adult). We used spat data to capture potential recruitment periods, and adult data to capture survival. Fall data capture responses of late spring and summer recruits in the spat category, and adult survival through the summer while winter data capture late fall recruitment, (spat), and recruitment from summer settlement to adult size (> 25 mm), along with adult survival.

### 2.3 Salinity Data

Daily interpolated salinity layers for the study area were developed for the years 2016–2020 following the methods used by Swam et al. (2022). We obtained empirical daily inshore salinity data from continuous data recorders maintained by the state of Louisiana Coastwide Reference Monitoring System (CRMS; CPRA 2021) and the US Geological Survey (USGS 2021). Daily offshore data were obtained from the Hybrid Coordinate Ocean Model (HYCOM) for salinity (GODAE 2021). HYCOM data were derived from remote sensing raster coverage and accessed through the data catalog of Google Earth Engine, an online computing platform for geospatial analysis using Google's infrastructure. From all sources, daily salinity means were obtained for January 1, 2016 through December 31, 2020. Point data outside the study area for the analysis were included, as available, to incorporate as much data as possible in the salinity interpolations. Salinity data were plotted and developed into rasters using Python scripting in ArcGIS v.10.8 and interpolated across the study area using the spline and barriers technique with a 500‐m resolution. Barriers included levees, impoundments, and basin boundaries affecting hydrologic flow to prevent interpolation across hydrologic boundaries (Swam et al. 2022). Interpolated values were validated against discrete salinity data taken from the Louisiana Department of Wildlife and Fisheries fishery‐independent monitoring data (LDWF 2018a; FisheriesDataDownload (la.gov)). We extracted interpolated values that matched location and day of the discrete values across coastal estuaries and years to explore their relationship using regression. Using these interpolated layers for each calendar year (2016–2020), we extracted total days and maximum continuous days of daily salinity means below thresholds of 1, 3, 5, 7, and 9 at each LDWF oyster station for the full calendar year, and for summer months (May 1–Aug 31), and binned them by salinity groups (0–1, > 1–3, > 3–5, > 5–7, > 7–9) for analyses, in order to ensure independence of variables.

### 2.4 Analyses

We explored two approaches to identify a changepoint across the salinity gradient and low salinity exposure for spat and adult oysters: RF and multilevel Bayesian models. The two statistical models were used to explore different operational definitions of thresholds, with RF providing insight into when abrupt changes in the relationship between our dependent variable, oyster abundance, and most influential salinity variables are predicted to occur, and Bayesian models identifying the salinity thresholds (level and exposure) that drive our dependent variable, oyster abundance, falling below a defined critical abundance threshold (< 50% of 5‐year mean), with more than 50% probability. Using both approaches, data were analyzed examining the fall oyster dredge abundance data in relation to the summer total and maximum continuous days salinity bins (*n* = 10 variables), and the winter oyster dredge data in relation to both summer and calendar year total and maximum continuous days salinity bins (*n* = 20 variables). Due to large differences in estuarine hydrology between Louisiana's western basins and eastern basins, we ran models separately by location (East: Pontchartrain + Barataria + Terrebonne; West: Atchafalaya + Vermilion + Calcasieu).

#### 2.4.1 Random Forest Model

Random forest (RF) modeling was selected as it provides a powerful tree‐based ensemble learning method. RF models enhance the prediction accuracy and reduce overfitting of individual trees by combining predictions from multiple trees (Breiman 2001). Like all the tree‐based methods, RF can (1) handle mixed type of predictors (e.g., categorical and numeric inputs); (2) capture the nonlinear effects on the response; (3) capture complex interaction effects among the input variables, and (4) have higher accuracy compared to other learning methods (Roubeix et al. 2016; Isabel et al. 2021; Rostami et al. 2021). We used the RF measure of variable importance plots, using increase in node purity (IncNodePurity) to determine relative variable importance, and partial dependence plots to examine the marginal effects of variables on the response (oyster abundance). Node purity is used to identify the variables that contribute most to reducing the homogeneity of the outcomes; variables that provide no information, or fail to explain outcomes, would have a low node purity score, and thus rank low in the variable importance plots. The partial dependence plots are then used to explore the direction and marginal effect of the top three variables identified. RF has a few hyperparameters, such as the number of trees included in the ensemble, the depth of the trees, and the number of inputs used for each split, but is generally less sensitive to choice of hyperparameters used than most machine learning models (Probst, Wright, and Boulesteix 2018).

Using the randomForest package in R (Code S1), and its default settings, which are generally recommended for these hyperparameters (Bergstra and Bengio 2012; Boulesteix et al. 2012), we explored the oyster and salinity variables identified above. Models with r‐square greater than 0.75 were kept for interpretation. Because we were interested in low salinity thresholds where our dependent variable, oyster abundance, showed a significant decline, we examined the top three predictor variables to identify the changepoint threshold. If the top three predictor variables were tightly grouped within the variable importance plot, we selected the lowest salinity bin; otherwise, we selected the most important variable for interpretation. Based on prior knowledge, we assumed any days of salinity falling within or above the identified salinity bin would also be positively associated with oyster abundance. The number of days when there was a large change in the marginal effect of the identified salinity bin was then used to calculate the temporal aspect of the threshold by subtracting this number from the total number of days possible (calendar year = 365 days; summer = 123 days). For example, if oyster abundance increased after 45 days of continuous summer salinity between 5 and 7 (salinity bin 5–7), we interpret a predicted decline in oyster abundance after 78 continuous days with salinity < 5. This interpretation is based on previous knowledge that salinity above 5 is generally not limiting to oysters, and so an indication of a large change in abundance after 45 days in the 5–7 bin likely reflects negative impacts from low salinity and are not due to days in the 7+ salinity bins.

#### 2.4.2 Multilevel Bayesian Models

We used Bayesian model approach to assess the same dataset to further explore identification of changepoints. Hierarchical Bayesian (HB) models allow for multiple sources of uncertainty by factoring complex relationships into conditional distributions in three stages (data, process model, and parameter) that can be used to draw inferences and make predictions (Clark 2005; Wu et al. 2012). The results are presented in posteriors that are readily summarized in quantiles and credible intervals to make probabilistic statements.

**FIGURE 2:**
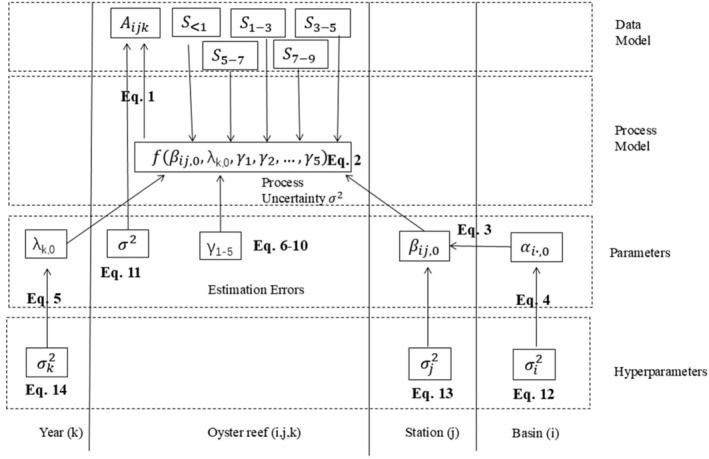
Hierarchical structure of a full model. The conceptual model illustrates the hierarchical structure of a full model (including all the spatial and temporal scales and all the covariates) for oyster abundance, with complexity decomposed into levels of data, process, and parameters along the vertical direction, and the association of different spatial and temporal scales along the horizontal direction. All the other models compared (Tables S1 and S2) are subsets of this full model. The equation numbers correspond to the equations shown in Figure S2 and in the Code S2 JAGS code. $A_{\mathrm{ijk}}$ denotes abundance at Station *j* in Basin *i* in Year *k*. $S_{<1},S_{1-3},S_{3-5},S_{5-7},\text{and}\;S_{7-9}$ denote annual or summer total number of days (or maximum of consecutive days) of salinity less than 1, 1–3, 3–5, 5–7, and 7–9, respectively, and *γ*_1‐5_ denote the coefficients associated with them. $\beta_{\mathrm{ij},0}$ denotes the intercept contributed by a station scale, which is nested within a basin and sampled from a parameter at the basin scale $\alpha_{i,0}$. *λ*_k,0_ denotes the intercept contributed by the annual scale. $\sigma^{2}$, $\sigma_{j}^{2}$, $\sigma_{i}^{2},\text{and}\;\sigma_{k}^{2}$ denote variances at the oyster reef, station, basin, and annual scales.

To account for the spatial and temporal correlation of the data that were clustered by station nested within basin, and across 5 years, we developed multilevel models. Traditional models such as ordinary regressions require the assumption of data independence that will be violated due to the nature of our data so are not appropriate tools for our study. The multilevel models explicitly model hierarchical data structures by including groups/scales which allow clustering of data within them (basin, station, or year). The groups are used for accounting for dependencies between the data (Schielzeth and Nakagawa 2013; Wu et al. 2018). We explored the following model scales: station nested within basin + year, station nested within basin only, basin + year, basin only, year only. The across‐scale/level/group information was associated using the intercept in the next‐level submodel, which means that the intercept varied by basin or year (Figure 2).

To represent the oyster abundance A at Station j in Basin i in Year k ($A_{\mathrm{ijk}}$), let $A_{\mathrm{ijk}}.\mu$ represents the mean of $A_{\mathrm{ijk}}$, and $\sigma^{2}$ represent the variance of A at the oyster reef scale. $A_{\mathrm{ijk}}$ was modeled by assuming it followed (~) a normal distribution (Equation 1):

(1) $$A_{\mathrm{ijk}}\sim N\left(A_{\mathrm{ijk}.}\mu ,\sigma^{2}\right)$$

The mean $A_{\mathrm{ijk}.}\mu$ was modeled as a linear function of the covariates at the oyster reef level: the annual or summer total number of days (or maximum consecutive days) of salinity less than 1 ($S_{<1}$), between 1 and 3 ($S_{1-3}$), between 3 and 5 ($S_{3-5}$), between 5 and 7 ($S_{5-7}$), and between 7 and 9 ($S_{7-9}$). (Equation 2):

(2) $$\begin{array}{c} A_{\mathrm{ijk}}.\mu =f\left(\beta_{\mathrm{ij},0,}\lambda_{k,0},\gamma_{1},\gamma_{2},\ldots ,\gamma_{5}\right)=\beta_{\mathrm{ij},0}+\lambda_{k,0}+\gamma_{1}\times S_{<1}\\ \;+\gamma_{2}\times S_{1-3}+\gamma_{3}\times S_{3-5}+\gamma_{4}\times S_{5-7}+\gamma_{5}\times S_{7-9} \end{array}$$

where $\beta_{\mathrm{ij},0}$ denotes the intercept contributed by a station scale, *λ*_*k*,0_ denotes the intercept contributed by the annual scale, and *γ*_1_, *γ*_2_, …, *γ*_5_ denote the coefficients of $S_{<1},S_{1-3},S_{3-5},S_{5-7},S_{7-9}$, respectively.

At the station scale, the intercept $\beta_{\mathrm{ij},0}$ is nested within basin, and was modeled by assuming it was distributed as (~) normal distributions:

(3) $$\beta_{\mathrm{ij},0}\sim N\left(\alpha_{i.,0},\sigma_{j}^{2}\right)$$

To complete the Bayesian model, we defined prior distributions for unknown parameters ($\alpha_{i.,0},\lambda_{k,0},\gamma_{1},\gamma_{2},\ldots ,\gamma_{5},\sigma^{2}$) and hyperparameters ($\sigma_{i}^{2},\sigma_{i}^{2},\sigma_{k}^{2}$). We used conjugated priors for computation efficiency (Calder et al. 2003), thus the priors and posteriors have the same probability distribution forms. The priors for $\alpha_{i.,0},\lambda_{k,0},\gamma_{1},\gamma_{2},\ldots ,\gamma_{5}$ (equations 4–10 in Figure S2) were normally distributed and the priors for $\sigma^{2},\sigma_{i}^{2},\sigma_{i}^{2},\sigma_{k}^{2}$ (equations 11–14 in Figure S2) followed the inverse gamma distributions. The priors' distributions were flat and only weakly influenced the posteriors, reflecting the lack of knowledge on the parameters (Lambert et al. 2005). We implemented the models in JAGS (Just another Gibbs Sampler) (Plummer 2003) and R using rjags package (Plummer 2024; Code S2).

We compared the full model (Figure 2) and subset models (Tables S1 and S2) based on deviance information criterion (DIC), and predictive posterior loss (PPL). The lower the DIC or PPL, the better the model predicts (Hooten and Hobbs 2015). DIC works well for comparing the models with the same hierarchical levels, however, we relied on PPL if the models showed different hierarchical levels. Once the model levels were determined, at the finest level, we modeled different variables of interest (spat or adult abundance in September–October—fall, or in November–December—winter, respectively), one at a time, as the linear functions of the maximum of continuous days or total number of days of salinity within the different bins identified in Table 1 in summer (May 1—August 31). Linear functions, though a simplification, could capture general trend of relations. In each model, we only considered one covariate. Then we compared the models with different covariates based on DIC to select the model(s) that best predicted the abundance of oysters in different life stages in fall and winter. The models with the difference of DIC of 2 (commonly used though there is no consensus among statisticians) are considered as similar models in prediction capabilities, therefore, we sometimes selected multiple best model candidates.

**TABLE 1:** Fall (Sep/Oct) and Winter (Nov/Dec) mean 2016–2020 oyster abundance (mean ± SD), range, and median by basin of spat (< 25 mm shell height) and adult (≥ 25 mm shell height) based on the Louisiana Department of Wildlife and Fisheries Fishery Independent Monitoring data (LDWF 2018; FisheriesDataDownload (la.gov), “Oyster Biological”) collected using a 24″ dredge.

	*n*	Fall		Winter	
		Spat	Adult	Spat	Adult
West Basins	118	10.3 ± 17.6 (0–95) 3.8	24.3 ± 26.3 (0–139.5) 14.6	7.5 ± 13.0 (0–61.5) 2.6	27.3 ± 28.1 (0–122) 18.5
Calcasieu	82	8.2 ± 13.2 (0–63.3) 2.6	26.3 ± 25.7 (0–139.5) 18.9	5.6 ± 9.3 (0–61.5) 2.3	31.8 ± 27.6 (0–122) 24.0
Vermilion‐Teche	25	16.0 ± 29.0 (0–95) 4.1	18.4 ± 25.5 (0.1–83.3) 9.5	5.3 ± 5.9 (0–19.5) 3.0	9.5 ± 11.3 (0–40.8) 6.3
Atchafalaya	11	13.2 ± 13.2 (1.3–37.8) 8.5	22.0 ± 32.7 (1–110.5) 13.9	25.2 ± 26.9 (0.5–61.5) 10.9	31.8 ± 41.0 (0.5–112) 14.0
East Basins	507	4.7 ± 11.6 (0–99.8) 0	10.4 ± 21.8 (0–119.4) 0	5.0 ± 15.2 (0–126.8) 0	8.2 ± 19.6 (0–132.8) 0
Terrebonne	108	17.9 ± 21.2 (0–99.8) 10.8	29.7 ± 32.3 (0–114.3) 15.3	23.8 ± 29.0 (0–126.8) 14.0	25.1 ± 32.7 (0–132.8) 13.8
Barataria	167	2.8 ± 4.6 (0–20.5) 0.6	20.0 ± 24.5 (0–116) 11.0	1.7 ± 3.5 (0–19) 0.3	14.6 ± 19.2 (0–71.5) 7.8
Pontchartrain	232	1.5 ± 4.8 (0–42.5) 0	2.0 ± 9.0 (0–119.4) 0	0.7 ± 2.6 (0–27.8) 0	1.5 ± 7.9 (0–110.5) 0

If the final models showed negative relations between variables of interest and number of days of a certain salinity level, we further ran the final models from 0 to 123 days (there is a total of 123 days from May 1 to August 31) to derive the median number of days at which a critical abundance threshold (defined as < 50% of the 5‐year mean) was reached. The derived number of days, either in total days, or maximum of continuous days of a certain salinity level, was interpreted as the threshold of days beyond which oysters could not tolerate this salinity level with more than 50% probability and is directly relevant to oyster management. If the final models showed positive relations between variables of interest and number of days of a certain salinity level, we further explored nonlinear relations using quadratic functions to evaluate whether they improved model predictions based on DIC. In addition, we evaluated the days of accumulated salinity (< 1, < 3, < 5, < 7, < 9), and further explored other salinity levels that presented negative relations even though DICs were larger than the best models.

## 3 Results

### 3.1 Oysters

Overall, mean spat and adult oyster abundance (oysters dredge^−1^) were approximately two–three times higher in west basins compared to east basins (Table 1). Across all samples, spat oyster abundance ranged from 0 to over 126.8 with lowest basin mean spat abundance reported in Pontchartrain winter (0.7 ± 2.6), and highest mean abundance reported for Atchafalaya winter (25.2 ± 26.9). Adult abundance ranged from a low of 0 to a high of 139.5, with lowest basin mean abundance in Pontchartrain winter (1.5 ± 7.9), while the highest was Calcasieu winter and Atchafalaya winter (31.8 ± 27.6, 31.8 ± 41.0, respectively). East basins had more than four times the number of oyster samples collected compared to west basins (*n* = 507 vs. 118), reflecting largely the greater areas of public oyster grounds compared to west basins.

### 3.2 Salinity

**FIGURE 3:**
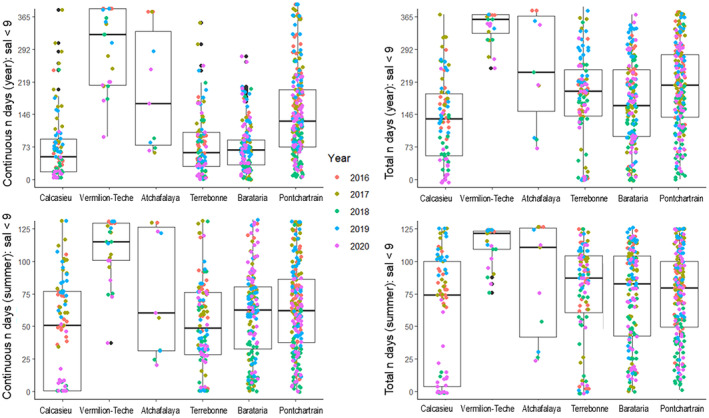
Daily salinity data stations and sources. Left column: Maximum number of continuous annual days (top) and continuous summer days (bottom) of salinity < 9, by basin and year. Right column: Total number of annual days (top) and total number of summer days (bottom) of salinity < 9, by basin and year. Annual = the calendar year (January 1–December 31, for 2016–2020). Summer = May 1—August 31; 2016–2020. Individual points represent individual stations within each basin. West = Calcasieu, Vermilion‐Teche, Atchafalaya; East = Terrebonne, Barataria, Pontchartrain.

**FIGURE 4:**
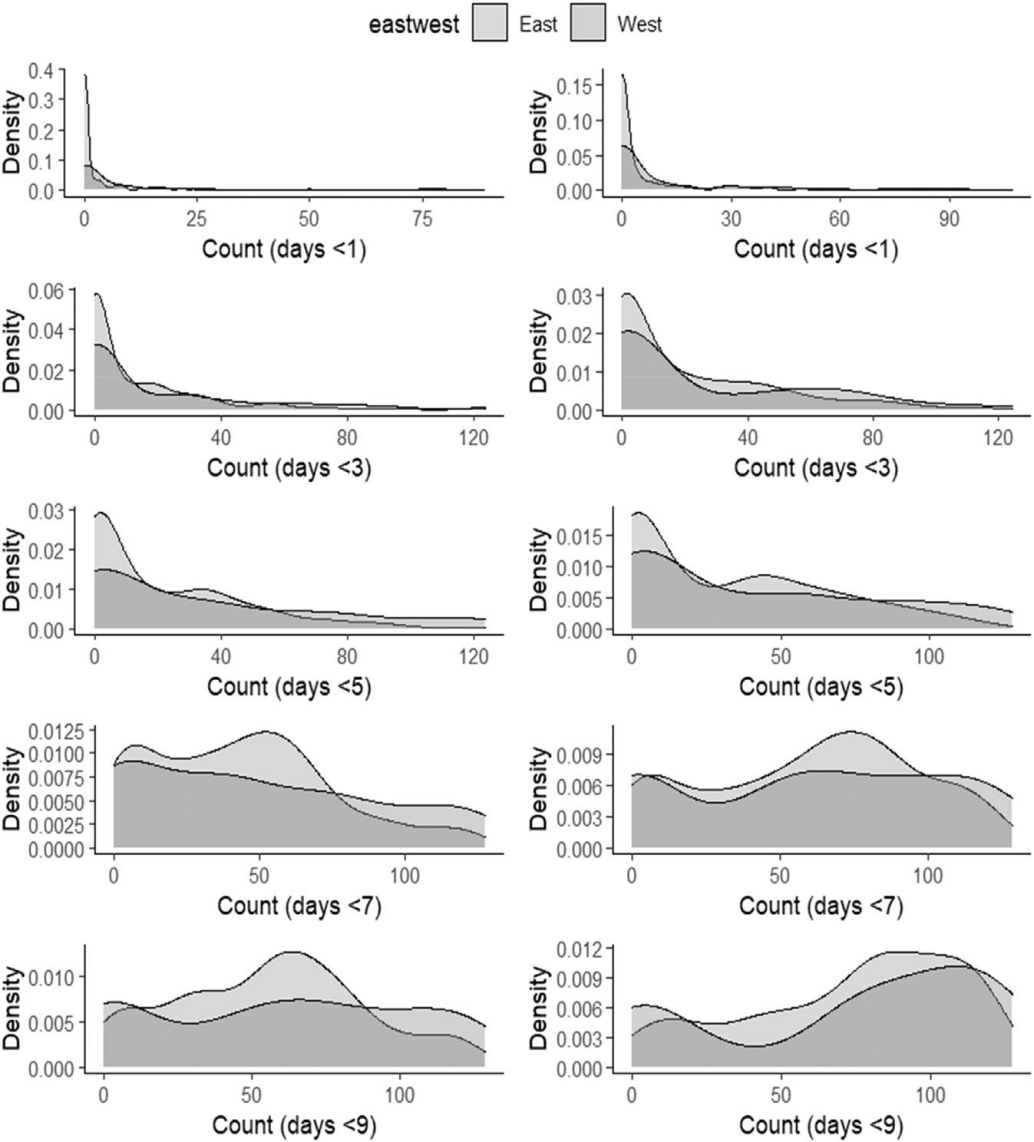
Salinity density plots. Density plots for east (Pontchartrain, Barataria, Terrebonne) and west (Calcasieu, Vermilion‐Teche, Atchafalaya) Louisiana basins for Left column: Summer maximum number of days of continuous salinity below a specific threshold (1, 3, 5, 7, 9), and Right column: Summer total number of days below a specific salinity level threshold (1, 3, 5, 7, 9) for 2016–2020.

Salinity measured during monthly oyster surveys ranged from 0.1 to 34.3 across all basins during 2016–2020 with basin means (±SD) ranging from a low of 6.2 ± 5.4 in the Vermilion basin to a high of 12.9 ± 5.9 in the Calcasieu basin (Table 2). Interpolated salinity values were validated against discrete salinity data across all basins and years (*n* = 3265; *r* ^2^ = 0.74). Using the interpolated daily salinity, all basins experienced on average, mean total and maximum continuous number of days of salinity less than 9 for more than 140 and 60 days, respectively, Figure 3). Many of these low salinity days occurred during the summer, where the mean total and continuous number of salinity days below 9 exceeded 75 and 45, respectively, across all basins. While exposure to salinity less than 9, 7 and 5 is consistently occurring, exposure to lower salinities below 3 and 1 while common, tend to occur less frequently, and in short periods, suggesting less frequent extended forays to these low salinities, but more acute exposure events (Figure 4; Figure S1).

**TABLE 2:** Mean salinity ± SD (range) of salinity by basin for 2016–2020 from the Louisiana Department of Wildlife and Fisheries Fishery Independent sampling (FisheriesDataDownload (la.gov), “Oyster Physical”).

Region	Basin	Mean ± SD (range)
West		11.4 ± 6.7 (0.1–30.2)
	Calcasieu	12.9 ± 6.0 (0.2–28.2)
	Vermilion‐Teche	6.2 ± 5.4 (0.1–26.6)
	Atchafalaya	9.4 ± 8.1 (0.1–30.2)
East		10.0 ± 6.6 (0.1–34.3)
	Terrebonne	11.7 ± 5.6 (0.1–26.9)
	Barataria	9.1 ± 5.9 (0.2–30.7)
	Pontchartrain	9.7 ± 7.2 (0.1–34.3)

### 3.3 Analyses

Models provided different results for salinity exposure thresholds, as described below, reflecting differences in the operational definition of a threshold implied for each approach (Table 3). RF results identified where abrupt changes in the relationship between the dependent variable (oyster abundance) and the independent variable (salinity) occurred; Bayesian models identified thresholds of salinity level and exposure that have more than 50% probability to drive oyster abundance below the critical abundance threshold, defined as less than 50% of the 5‐year mean abundance.

**TABLE 3:** Summary of identified salinity exposure thresholds identified using multilevel Bayesian modeling and Random Forest, by oyster life stage (spat: < 25 mm shell height; adult: ≥ 25 mm shell height) and season (Fall: September–October; Winter: November–December) and region (E, east; W, west) in Louisiana, USA. Salinity identifies the salinity level identified for the threshold; Exposure identifies the timing (i.e., summer), and whether days must be continuous, or just total counts. For Bayesian models, only negative models with either 95% credible intervals (CI) (bold) or 50% CI are reported; “—” indicates other (positive for fall adult abundance in the east, weak negative for fall and winter adult abundance in the west). Bayesian models identified thresholds beyond which more than 50% of probability that oyster abundance was below the critical abundance threshold, defined as less than 50% of the 5‐year mean abundance. Random forest results are presented for models with *r* ^2^ > 0.75 and interpreted using the variable importance, and partial dependence plots, where abrupt changes in the partial dependence plots were used to identify critical salinity exposure thresholds.

Abundance response variables		Region	Multilevel Bayesian models		Random Forest	
Life stage	Season		Salinity	Exposure	Salinity	Exposure
**FallE< 585 continuous summer days< 793 continuous summer days** Spat	Fall	E	< 5	85 continuous summer days	< 7	93 continuous summer days
	Winter	E	—	—	< 3	83 continuous summer days
Adult	Fall	E	**< 1**	**20–90 continuous summer days**	< 7	95 continuous summer days
	Winter	E	< 1	10–80 continuous summer days	< 7	87 continuous summer days
Spat	Fall	W	< 3	> 123 summer days	< 1	60 total summer days
	Winter	W	< 3	100–115 total summer days	< 1	60 total summer days
Adult	Fall	W	—	—	< 5	58 continuous summer days
	Winter	W	—	—	< 5	58 continuous summer days

#### 3.3.1 Random Forest Models

All models had *r* ^2^ values greater than the 0.75 threshold. For winter oyster abundance models, comparison of models with all calendar year and summer salinity variables (20 variables) to models with only summer salinity variables (10 variables) demonstrated only marginal model improvements, so calendar year variables were removed from final models. Using partial dependence plots, the identified predictor variables were interpreted as identifying positive thresholds; we assumed salinity below the identified thresholds was associated with decreased predicted abundances (Figures S3 and S4).

**FIGURE 5:**
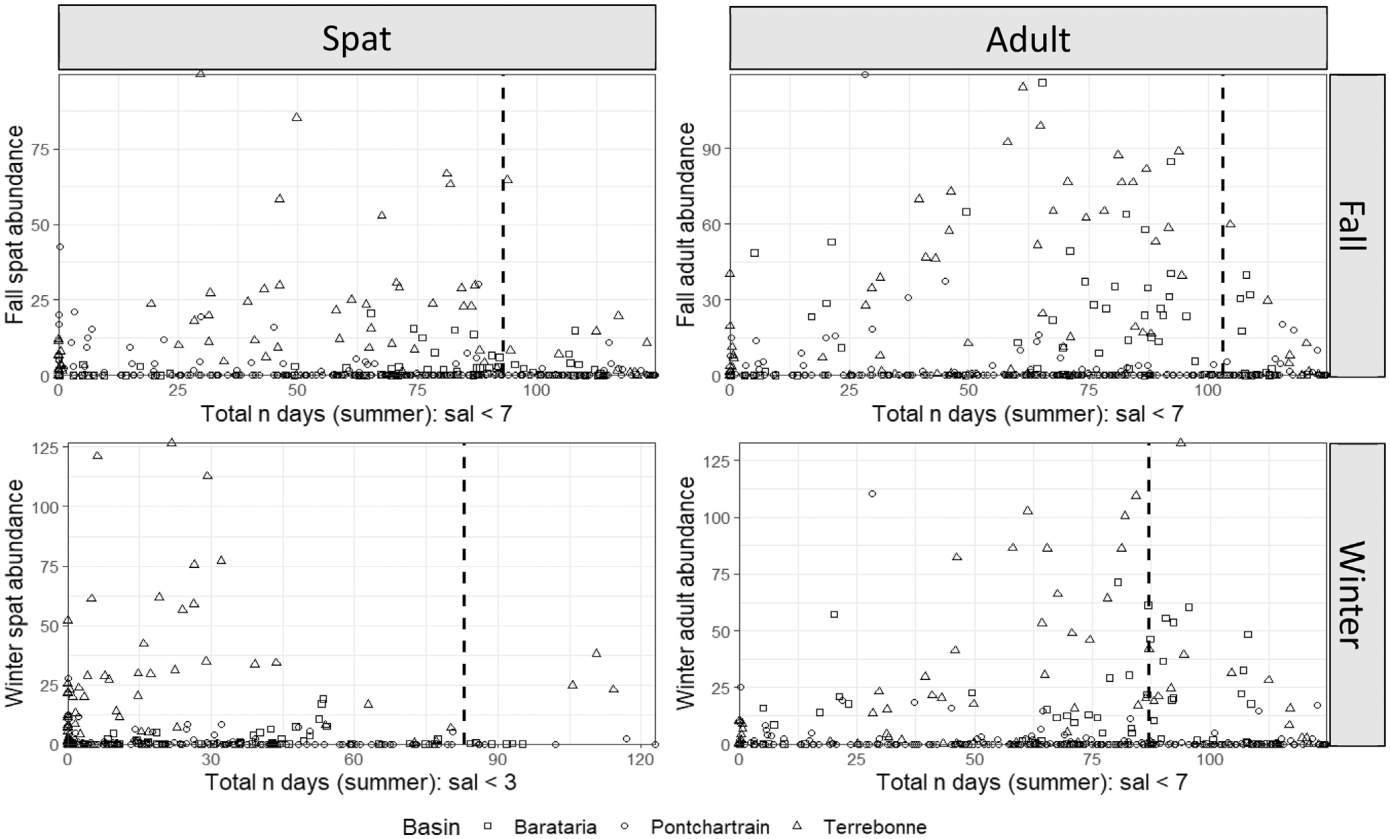
East Basin Random Forest. East Basins (Pontchartrain + Barataria + Terrebonne basins) variable importance (left column) and partial dependence (right column) plot of variables identified for interpretation as critical lower salinity threshold oyster spat (< 25 mm shell height) or adult (≥ 25 mm shell height) abundance during fall (September/October) or winter (November/December) monitoring completed by the Louisiana Department of Wildlife and Fisheries during their long‐term monitoring collection. Variable selected was either the variable of highest importance, or, when the top three variables were clustered in importance, the lowest salinity range of the top three variables. Variables explored included the total number (s) or the maximum continuous number (c) of days where salinity was within summer (su) salinity bins of < 1, 1 to < 3, 3 < 5, 5 < 7, and 7 < 9, and are coded such that the variable for total number of days of salinity between within the 7 < 9 bin is s_d_su7.9, or maximum continuous number of days within the 7 < 9 bin is c.d_su7.9.

All four models for east basins identified a mix of continuous and total salinity days as the top three predictor variables (Figure 5). The fall model for spat indicates that spat abundance increases when total number of days of salinity of 7–9 increases from 15 to 30 days and is stable when total number of days of salinity 7–9 are greater than 30 (Figure 5). This indicates that a potential threshold exists whereby spat abundance is limited when total number of days of salinity < 7 is above 93 (123 days in summer—30 days). The spat winter model had two variables of high importance, total number of days between 7–9, and between 3–5 with both indicating an increase in spat abundance with more days within those salinity bins. The partial dependence plot for 3–5 shows increasing spat abundance when number of total days increases from 30 to 40, and then predicts stable spat abundance after this threshold. This indicates a potential threshold whereby spat abundance is limited when total number of days of salinity < 3 exceeds 83 (123–40). Both the adult fall and winter models indicate that predicted adult abundance increases when total number of days of salinity of 7–9 increases (Figure 5). The fall model indicates increasing adult abundance when total number of days of salinity of 7–9 increases between 15 and 20 days, and stable after that while the winter model indicates increasing adult abundance when total number of days of salinity increases between 10 and 35 days and is stable after that. These suggest a potential threshold for fall of 103 days (123–20), and for winter of 87 days (123–35) of salinity < 7 for adult abundance.

**FIGURE 6:**
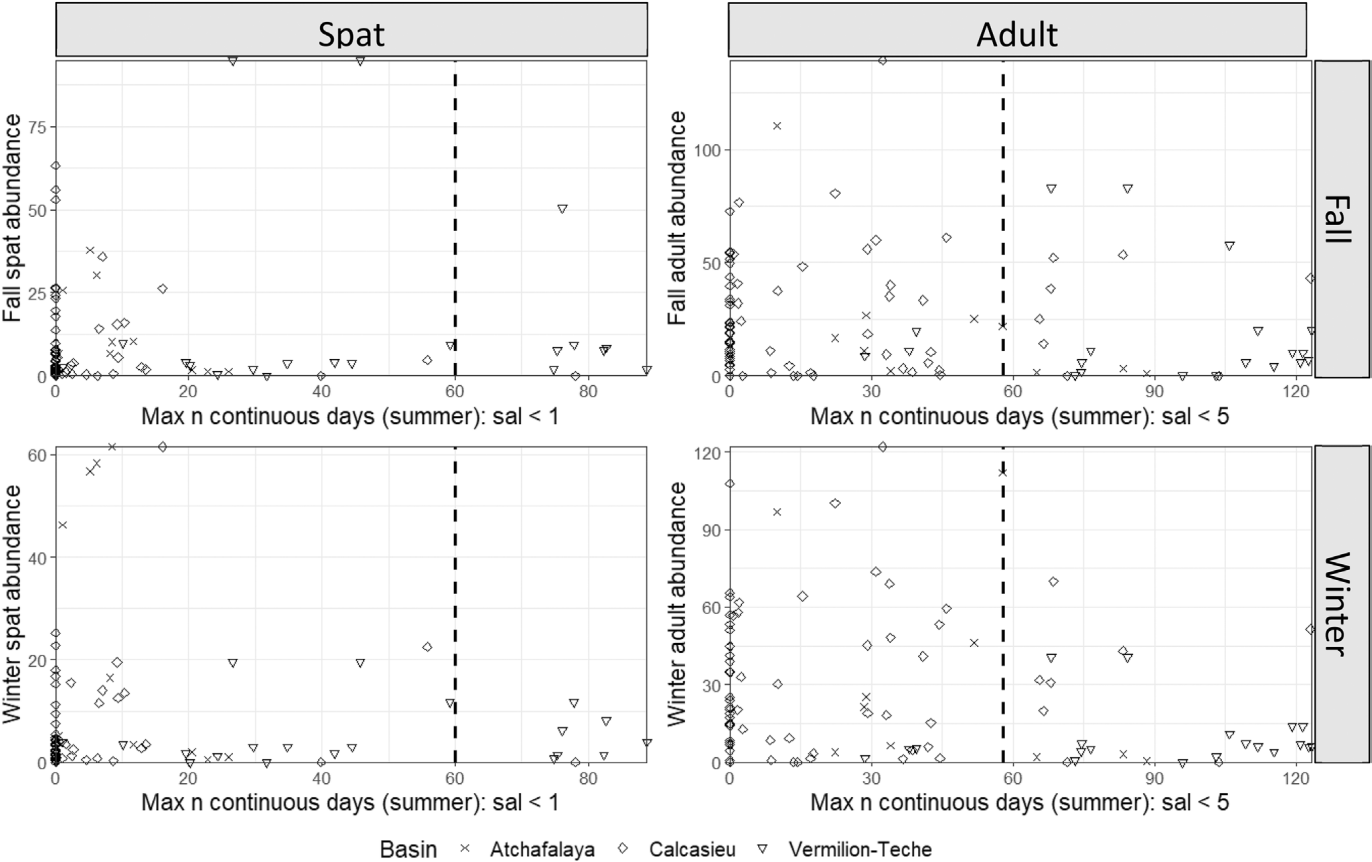
West basin random forest. West Basins (Calcasieu, Vermilion‐Teche, Atchafalaya) variable importance (left column) and partial dependence (right column) plot of variables identified for interpretation as critical lower salinity threshold oyster spat (< 25 mm shell height) or adult (≥ 25 mm shell height) abundance during fall (September/October) or winter (November/December) monitoring completed by the Louisiana Department of Wildlife and Fisheries during their long‐term monitoring collection. Variable used for importance was either the variable of highest importance, or, when the top three variables were clustered in importance, the lowest salinity range of the top three variables. Variables explored included the total number (s) or the maximum continuous number (c) of days where salinity was within summer (su) salinity bins of < 1, 1 to < 3, 3 < 5, 5 < 7, and 7 < 9, and are coded such that the variable for total number of days of salinity between within the 7 < 9 bin is s_d_su7.9, or maximum continuous number of days within the 7 < 9 bin is c.d_su7.9.

All four models for west basins identified a mix of continuous and total salinity days as the top three predictor variables (Figure 6). The spat fall and winter models indicate that spat abundance increases when total number of days of salinity < 1 ranges from 10 to 60, but then decreases after more than ~60 days. These models suggest a potential threshold of decreased spat abundance when more than 60 days had salinity levels below 1 (Figure 6). Similarly, both the adult fall and winter models indicate that predicted adult abundance increases when continuous number of days of salinity of 5–7 increases between ~15 and 65 days; there are few data points to inform the model after 65 days. These models suggest a potential threshold of continuous days of salinity < 5 of about 58 days (123–65) where predicted adult abundance would be expected to decrease.

#### 3.3.2 Multilevel Bayesian Models

We selected the best models and identified the most important covariates for each variable of interest in east and west regions, respectively (Table 4; Table S1). There was a mix of positive and negative relations found in the models (Table S2). When there was a positive relation in the model, we further explored the quadratic functions, but in all cases, they failed to improve any model predictions. The best model candidates that indicated negative relations between the covariates and response variables were used to make predictions and identify thresholds of days beyond which the predicted abundance dropped below the critical abundance threshold with more than 50% of probability, as defined in methods.

**TABLE 4:** Final Bayesian models selected for each variable of interest by oyster life stage (spat: < 25 mm shell height; adult: ≥ 25 mm shell height) and season (fall: September–October; Winter: November–December) and region (east; west) in Louisiana, USA. Bold models are retained for analyses. Spatial and temporal levels reflect model levels of sample station (S), estuarine basin (B, Figure 1), and year (Y; 2016–2020). Covariates count of either maximum continuous days (M) or total number of days (T) of salinity during the summer (May 1–August 31) corresponding to the salinity level or bin identified. Results indicate whether the relationship identified is negative (−) or positive (+), while probability is the probability that either 95% of credible interval (CI) of coefficient for the selected covariate does not overlap 0, or 50% of probability indicates that 50% of CI does not overlap 0, while 95% of CI does, and “—” indicates that both 50% and 95% of CIs overlap 0. Results with two models are presented if the differences in deviance information criterion are less than 2 (Table S2).

Response variables		Region	Spatial and temporal levels	Covariates		Results	
Life stage	Season			Salinity	Count	Median	Probability (%)
Spat	Fall	**E**	**S within B + Y**	**< 5**	**M**	**−**	**95**
	Winter	E	S within B + Y	5–7	M	+	95
Adult	Fall	**E**	**S within B + Y**	**< 1**	**M**	**−**	**95**
	Winter	E	S within B + Y	7–9	M	+	50
		**E**	**S within B + Y**	**< 1**	**M**	**−**	**50**
Spat	Fall	W	S within B + Y	7–9	T	+	95
		**W**	**S within B + Y**	**1–3**	**T**	**−**	**50**
	Winter	**W**	**S within B + Y**	**1–3**	**T**	**−**	**50**
Adult	Fall	W	S within B + Y	5–7	M	+	—
		W	S within B + Y	< 1	M	−	—
	Winter	W	S within B	3–5	M	−	—

**FIGURE 7:**
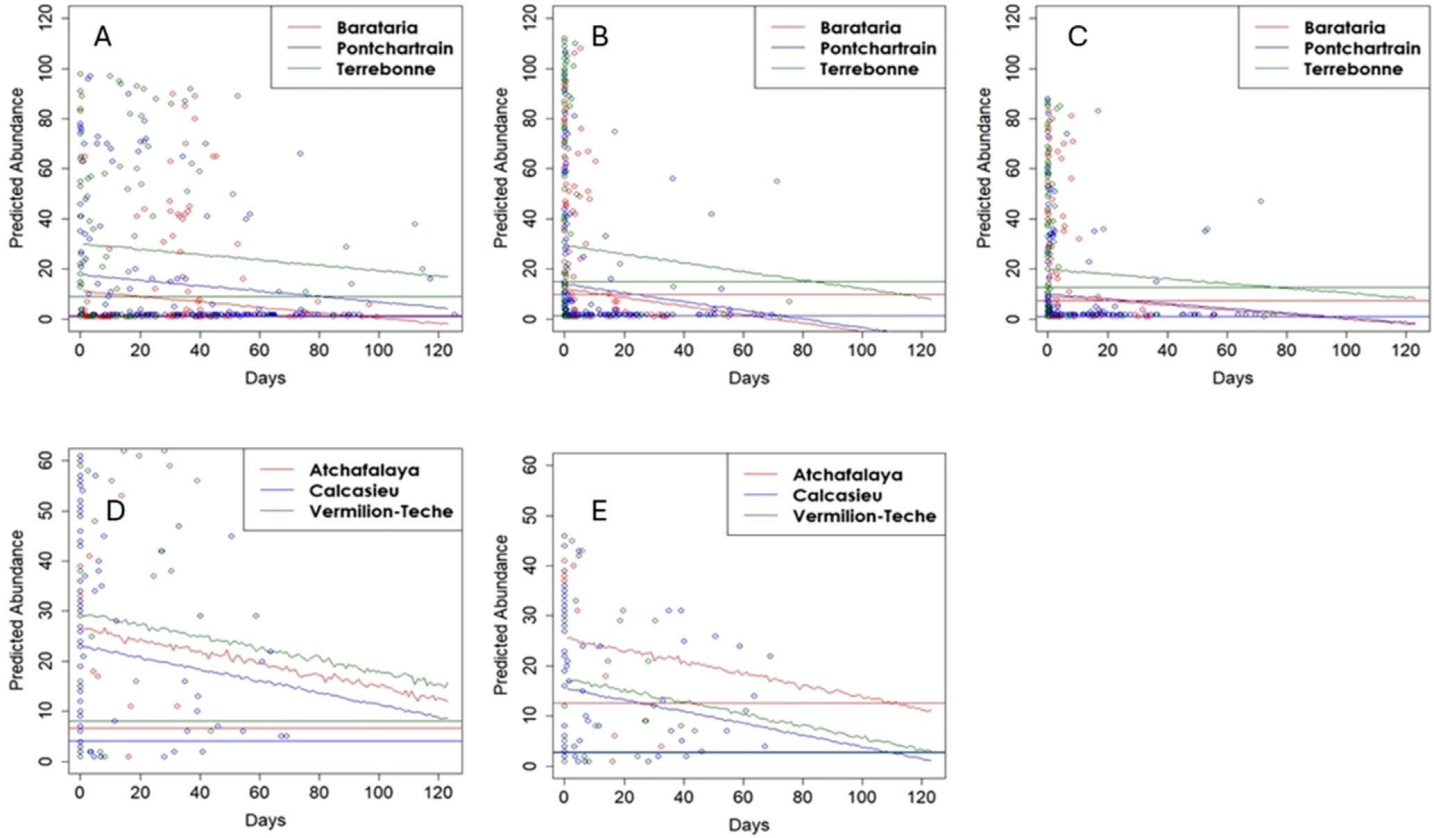
Top row: Significant negative east basin models identified using multilevel Bayesian models of predicted oyster abundance medians versus the total days of salinity for (A) fall spat (maximum of continuous days of salinity less than 5 in summer), (B) fall adult (maximum of continuous days of salinity less than 1 in summer), and (C) winter adult (maximum of continuous days of salinity less than 1 in summer). Bottom row: Significant negative west basin models identified using multilevel Bayesian models of predicted oyster abundance medians versus the total days of salinity between 1 and 3 in summer, in reference to the critical abundance predefined as the half of the 5‐year average in each basin in the western region for (D) fall spat, and (E) winter spat. Threshold number of days occurs where the model trend line crosses the horizontal line indicating a 50% level of the 5‐year average in oyster abundance (critical abundance). No other models met our criteria for negative relations or showed a significant impact on abundance.

The best models for east basin adult and spat in fall and winter included station nested within basin + year (Table 4). The fall spat and adult, and winter adult models identified negative relationships between predicted abundance and maximum continuous salinity. The threshold number of days occurs where the model trend line crosses the horizontal line indicating a 50% level of the 5‐year average in oyster abundance (critical abundance). The positive model relationships suggest that thresholds (days) of certain low salinity levels could not be detected by the Bayesian approach although the low salinity magnitude is detected by the method. These models indicate that predicted oyster abundance would fall below the critical abundance threshold as the maximum number of continuous days of salinity < 5 (spat) or < 1 (adult) increased. Temporal thresholds (number of days of identified salinity levels beyond which the predicted abundance falls under the predefined critical values with more than 50% of probability) differed by life stage, season, and basin (Figure 7).

For east basin models temporal threshold results for spat abundance differed by season. While the number of continuous days of salinity < 5 was the most informative covariate to predict the fall‐season abundance (negative relation), the number of days of salinity between 5 and 7 predicted the winter‐season abundance the best (positive relation). For fall spat, model results indicate that abundance for Barataria Basin would fall below the critical abundance threshold when more than 85 days were continuously below 5 in summer. While the relationships for the other two basins were negative, the predicted median abundance did not fall below the identified critical abundance threshold. For adult abundance, the continuous number of days of salinity < 1 was selected as the best models for both seasons with a decrease in adult abundance with an increasing number of continuous days of salinity below 1. Models predicted that adult abundance would have a greater than 50% probability of falling below the critical abundance threshold with continuous days of salinity below 1 exceeding an estimated 20 (Barataria), 90 (Pontchartrain), or 80 (Terrebonne) days for fall abundance, and an estimated 10 (Barataria), 70 (Pontchartrain), or 80 (Terrebonne) days for winter abundance.

For west basins, the best models for fall and winter spat and fall adult included station nested within basin + year, and for winter adult, station nested within basin (Table 3). The fall and winter spat models identified a negative relationship, with predicted oyster abundance falling below the critical abundance threshold as the total number of days of salinity from 1 to 3 increased. Temporal thresholds differed by basin, although all indicated a threshold greater than 90 days before predicted medians would cross the 50% abundance threshold (Figure 7D,E). The best models for adult abundance selected number of days of salinity less than 1 for the fall, and between 3 and 5 for the winter. In both cases, while the medians of the coefficients were negative, they did not show a significant impact on abundance as 50% of credible intervals of their coefficients overlapped 0, suggesting that temporal thresholds of low salinity (< 1 or < 3) could not be detected.

## 4 Discussion

Using the eastern oyster as a case study, this work highlights the complexity and multiple dimensions defining thresholds and provides valuable data to consider for management of this economically and ecologically important species. Regardless of life stage or location, summer salinity regimes of continuous or total days of salinity below a certain level were consistently identified as predictive of abrupt changes in oyster abundance (i.e., RF) or oyster abundance declines below a critical abundance threshold (< 50% of 5‐year mean; i.e., Bayesian models). A few key lessons learned include (1) Louisiana oysters are robust to short‐term low salinity exposure (< 5), but there is a strong seasonal effect with lower robustness to exposure to low salinity during summer (higher temperature, reproductive period); (2) thresholds differ by oyster life stage and location; (3) similar to experimental field and laboratory studies, low salinity thresholds are defined by multiple dimensions (e.g., level, duration, timing; La Peyre et al. 2003, 2013; McFarland, Rumbold, et al. 2022; McFarland, Vignier, et al. 2022); and (4) results differed by statistical approach reflecting the different operational definition of thresholds implied based on the analyses, which is rarely acknowledged. These findings underline the challenge of defining thresholds and identify the need to consider carefully the appropriate relevant threshold to be considered when exploring the concept of critical thresholds and applying them for management.

This work affirms observations over the last 100 years of oyster exposure and tolerance to frequent and extended low salinity events in key oyster growing areas (Butler 1952, 1954; Loosanoff 1952; Andrews, Haven, and Quayle 1959; Cake 1983; Chatry, Dugas, and Easley 1983; La Peyre et al. 2003, 2013; La Peyre, Gossman, and La Peyre 2009; Beseres Pollack et al. 2011; Lowe et al. 2017). Specific critical salinity exposure threshold levels identified (Table 3) support the contention that Louisiana oysters are relatively tolerant to extended forays to low salinity, but that there is a synergistic effect of high temperatures on their salinity tolerance. Declines in oyster abundance were consistently predicted by low salinity occurrence during the summer months (May–August). The mean salinity measured during oyster abundance collection used in this study ranged from a mean of 6.2–12.9 across the public oyster grounds (Table 2). These salinity means are below what is generally reported as optimum for oyster growth and survival and considered nonoptimal within habitat suitability models for Louisiana (i.e., Lindquist et al. 2021). Lowe et al. (2017) noted through an analysis of 20 years of Louisiana data that the optimal salinity range for growth and survival for Louisiana oysters was lower than generally reported for the species, ranging from 11 to 16. Lowe et al. (2017) also found that as the percentage of days of salinity < 5 increased, oyster growth and survival decreased, although most of these low salinity days occurred during hotter temperatures.

Declining oyster abundance in response to low summer salinity matches the well‐documented synergistic effects of reduced tolerance to low salinity when exposed to warmer temperatures, and for adults, during gametogenesis (Loosanoff 1952; Andrews, Haven, and Quayle 1959; Heilmayer et al. 2008; Marshall, Casas, et al. 2021; Marshall, Coxe, et al. 2021; McFarland, Rumbold, et al. 2022). Summer water temperatures in coastal Louisiana average over 28°C, and regularly exceed 30°C, with forays as high as 34°C (Lowe et al. 2017). Several oyster studies have documented high tolerance to low salinity events during low (winter) temperatures in the field, with tolerances to salinity < 5 less lethal during winter and spring periods as compared to during summer periods (Butler 1952; Andrews, Haven, and Quayle 1959; La Peyre et al. 2013; McFarland, Rumbold, et al. 2022). This synergistic effect of temperature and salinity is also highlighted by a recent laboratory study where Louisiana oysters experienced less than 15% mortality after 3 months at a salinity < 3 when held at a moderate temperature of 25°C (Marshall, Coxe, et al. 2021) but experienced over 75% mortality within 3 weeks at a salinity of 4, and temperature of 33.3°C (Marshall, Casas, et al. 2021; Marshall, Coxe, et al. 2021). In addition, the potential contributing effects of gametogenesis, which tends to coincide with the summer period in Louisiana may also contribute to reduced oyster survival and decreased adult abundances (Butler 1949; Andrews, Haven, and Quayle 1959; McFarland, Rumbold et al. 2022; Gregory, McFarland, and Hare 2023).

Defining critical thresholds for low salinity tolerance of oysters is complicated by ontogeny (Loosanoff 1952; Lowe et al. 2017; Pruett et al. 2021; McFarland, Vignier, et al. 2022). Field and laboratory studies demonstrate size class‐dependent vulnerability to salinity and temperature extremes, with larger (i.e., adult) organisms generally more sensitive to extreme conditions as compared to smaller (spat) organisms (Peck et al. 2009; Munroe et al. 2013; Rybovich et al. 2016; McFarland, Vignier, et al. 2022). Although not explored in this study, gametes, embryos, and larvae are less tolerant to salinity and temperature compared to settled oysters, which may significantly impact spat abundances in the field (Davis and Calabrese 1964; Hayes and Menzel 1981; Shumway 1996). In addition, a controlled study confirmed the synergistic effects of higher temperatures on low salinity but demonstrated that the synergistic effects increased as oysters got larger (i.e., larvae < spat < adult), suggesting that with more summer temperature extremes, low salinity events may become more lethal to adults (McFarland, Vignier, et al. 2022). Here, spat were generally found to have lower salinity thresholds as compared to adults (i.e., salinity < 1 for spat vs. < 5 for adults), however, spat abundances are likely impacted by adult abundance and condition as much as larval and spat settlement and survival.

Past studies suggest salinity less than 10 results in depressed gametogenesis, delayed spawning, disintegration of gonads, or high postsettlement mortality (Butler 1949; Loosanoff 1952; Davis and Calabrese 1964; Livingston et al. 2000), with studies from the Gulf of Mexico specifically identifying salinity below 10 as indicative of limited or no recruitment (Cake 1983; Chatry, Dugas, and Easley 1983). The abundance data analyzed here indicate spat at salinities well below 10; this could be from recruitment from more distant reef areas where salinity remained high as larval transport may result in long‐distance movement of larval resources (i.e., North et al. 2008; Kim et al. 2010; Swam et al. 2022), or reproduction occurring prior to lowered salinities, and larval and postsettlement survival occurring. However, we lack information to predict oyster fecundity, larval dispersal, and settlement (Mroch III, Eggleston, and Puckett 2012; Glandon et al. 2016; Marshall et al. 2020; McFarland, Rumbold, et al. 2022; McFarland, Vignier, et al. 2022). Without associated gametogenesis data to track adult reproduction, it is difficult to tease out the role of reproductive output, larval survival, or postsettlement survival in explaining observed patterns in spat abundances, or to predict their recruitment to the adult population. Salinity exposure threshold differences between east and west basin oyster models may also reflect variation due to location effects, which could reflect recruitment variation, phenotypic and genotypic variation, and the impacts of prior exposures in oyster populations (Eierman and Hare 2013, 2015; Leonhardt et al. 2017; Sehlinger et al. 2019; Sirovy et al. 2023).

Thresholds identified were multidimensional in that they were defined by both a specific salinity level, and an exposure (continuous versus total, length of time). Continuous exposure to low or stressful salinity levels is likely more limiting as short‐term forays into stressful conditions are not on their own lethal to oysters, as they can seclude themselves from the environment by closing their valves (Loosanoff 1952; Casas et al. 2018). However, long‐term survival when exposed to lowered salinity depends on the oysters' ability to osmoconform, as long‐term valve closure can result in mortality from the accumulation of waste products from the anaerobic metabolism, and reduction in feeding (Loosanoff 1952; de Zwaan and Wijsman 1976; Sokolova et al. 2012). McFarland, Vignier, et al. (2022) found that adult oyster survival when exposed to low salinity, was significantly improved when they also experienced short‐term forays to higher salinity. Casas et al. (2018) found that Louisiana oysters at salinity of 3 showed less valve opening and feeding as compared to oysters at higher salinity, and a follow‐up study demonstrated that oysters osmoconformed faster at higher temperatures (28°C vs. 23°C) when salinity was ≤ 1.5, but also experienced more rapid mortality. Other field and laboratory studies using Louisiana oysters consistently find minimal plasma osmolality of about 80 mOsm kg^−1^, which is equivalent to a salinity of about 2.5 when exposed to lower salinity levels (La Peyre et al. 2003; La Peyre, Gossman, and La Peyre 2009; Casas et al. 2023). Closure of valves for extended periods of time also reduces food intake, which can result in decreased condition, and increased mortality over time (Casas et al. 2018).

Increasing probabilities of extreme precipitation events and heatwaves enhance the likelihood of more frequently crossing salinity exposure thresholds impacting species distributions and abundances (Jentsch, Kreyling, and Beierkuhnlein 2007). Early work suggested the concept of “killing floods” based on long‐term salinity means (Cake 1983). This early definition was incorporated into early habitat suitability models and defined as a 3‐month salinity average of ≤ 2 (Soniat and Brody 1988), with more recent models refined using a 1‐month summer average of ≤ 2 (Lindquist et al. 2021). Here, results suggest low salinity exposure thresholds in Louisiana estuaries are defined as the number of continuous salinity days below 5 ranging from 10 to over 100, depending on the life history stage and the basin. Numerous models seek to identify this salinity exposure threshold however, a fair bit of uncertainty still exists; in using field data, identification of explicit salinity regimes (intensity, duration) remains challenging as other potential factors might influence the collected abundance data. These other factors might include rate of salinity change, low dissolved oxygen levels, infection by pathogens, predation, potential toxins, tropical storms and hurricanes, and harvest (La Peyre et al. 2003, 2013; Coxe et al. 2023; McFarland, Rumbold et al. 2022). The oyster abundance data used in these analyses were taken from long‐term monitoring on Louisiana's public oyster grounds, however, less than 5% of total harvest reported was taken from these areas during the years 2016–2020 (LDWF 2016, 2017, 2018b, 2019, 2020). In addition, most of the other potential stressors are not routinely measured across significant areas of Louisiana waters, and their occurrences across oyster areas are not well documented but future investigations could help clarify their potential impacts and synergies with low salinity events.

This work highlights the complexity and multiple dimensions required to consider when exploring thresholds impacting population resilience. In particular, while thresholds are generally explored as a means to identify a “tipping point,” considered to be a point from which a resource may not recover, the search for a universal critical threshold may hold false promise (e.g., Hillebrand et al. 2020). Multiple drivers, life stage, spatial context, local adaptations, past disturbances, and defining actual “tipping points” of ecosystems, or species complicate the identification and discussion surrounding thresholds or tipping points (Hiddink and Kaiser 2005; Truebano et al. 2018; Hillebrand et al. 2020; Carrier‐Belleau et al. 2023). At the same time, acknowledging this complexity highlights the need to better understand mechanistic responses and drivers of species (Hillebrand et al. 2020; Dudney and Suding 2020). For the eastern oyster, physiological laboratory studies helped interpret the results by demonstrating how past exposure, interacting factors (i.e., temperature, dissolved oxygen), and stressor dimensions (i.e., timing, rate of change, variability) impact oysters lethally, and sublethally; future research for identification of ecological thresholds would benefit from better integration of these experimental studies. For the eastern oyster, understanding the key stressors that impact oyster population abundances can help guide management and restoration approaches, by informing site selection to ensure metapopulation connectivity across areas that may cross thresholds. In Louisiana estuaries, the management of river diversions to avoid extended low salinity events during summer months could be prioritized. Identifying thresholds to inform management requires acknowledging the potentially important multiple dimensions of drivers, interacting drivers, and incorporating mechanistic understanding to interpret ecological impacts and understand underlying causes of change.

## Author Contributions

**Megan K. La Peyre:** conceptualization (lead), data curation (equal), formal analysis (equal), funding acquisition (lead), investigation (equal), methodology (equal), project administration (lead), resources (equal), supervision (lead), visualization (equal), writing – original draft (lead), writing – review and editing (equal). **Hongqing Wang:** conceptualization (equal), formal analysis (equal), investigation (equal), methodology (equal), validation (equal), visualization (equal), writing – review and editing (equal). **Shaye E. Sable:** conceptualization (equal), data curation (lead), investigation (equal), methodology (equal), validation (equal), visualization (equal), writing – review and editing (equal). **Wei Wu:** formal analysis (equal), investigation (equal), methodology (equal), writing – review and editing (equal). **Bin Li:** formal analysis (equal), investigation (equal), methodology (equal), validation (equal), writing – review and editing (equal). **Devin Comba:** data curation (equal), visualization (equal), writing – review and editing (equal). **Carlos Perez:** data curation (supporting), methodology (equal), visualization (supporting). **Melanie Bates:** data curation (supporting), methodology (supporting), visualization (supporting). **Lauren M. Swam:** formal analysis (equal), methodology (equal), visualization (equal), writing – review and editing (equal).

## Conflicts of Interest

The authors declare no conflicts of interest.

## Supporting information


Data S1.

## Data Availability

The data that support the findings of this study are openly available from Coastal Protection and Restoration Authority of Louisiana at https://cims.coastal.louisiana.gov, Global Ocean Data Assimilation Experiment at https://code.earthengine.google.com/ee.ImageCollection, National Oceanic and Atmospheric Administration, https://code.earthengine.google.com/ee.ImageCollection (NOAA/CDR?OISST/V2_1), and U.S. Geological Survey.

## References

[ece370759-bib-0001] AghaKouchak, A. , F. Chiang , L. S. Huning , et al. 2020. “Climate Extremes and Compound Hazards in a Warmining World.” Annual Review of Earth and Planetary Sciences 48: 519–548. 10.1146/annurev-earth-071719-055228.

[ece370759-bib-0002] Andrews, J. D. , D. Haven , and D. B. Quayle . 1959. “Fresh‐Water Kill of Oysters ( *Crassostrea virginica* ) in the James River, Virginia 1958.” Proceedings of the National Shellfisheries Association 49: 29–49.

[ece370759-bib-0003] Bayne, B. 2017. Biology of Oysters ed. 1. Vol 41. London: Academic Press.

[ece370759-bib-0004] Bergstra, J. , and Y. Bengio . 2012. “Random Search for Hyper‐Parameter Optimization.” Journal of Machine Learning Research 13: 281–305.

[ece370759-bib-0005] Beseres Pollack, J. , H.‐C. Kim , E. K. Morgan , and P. A. Montagna . 2011. “Role of Flood Disturbance in Natural Oyster ( *Crassostrea virginica* ) Population Maintenance in an Estuary in South Texas, USA.” Estuaries and Coasts 34: 187–197.

[ece370759-bib-0006] Bindoff, N. L. , W. W. L. Cheung , J. G. Kairo , et al. 2019. “Chapter 5: Changing Ocean, Marine Ecosystems, and Dependent Communities.” Intergovernmental Panel on Climate Change. In IPCC Special Report on the Ocean and Cryosphere in a Changing Climate, pp. 447–587.

[ece370759-bib-0007] Boulesteix, A. L. , S. Janitza , J. Krupp , and I. R. Konig . 2012. “Overview of Random Forest Methodology and Practical Guidance With Emphasis on Computational Biology and Bioinformatics. WIREs.” Data Mining and Knowledge Discovery 2: 493–507.

[ece370759-bib-0008] Breiman, L. 2001. “Random Forests.” Machine Learning 45, no. 1: 5–32.

[ece370759-bib-0009] Butler, P. A. 1949. “Gametogenesis in the Oyster Under Conditions of Depressed Salinity.” Biological Bulletin 96: 263–269. 18153114

[ece370759-bib-0010] Butler, P. A. 1952. “Effect of Floodwaters on Oysters in Mississippi Sound in 1950.” Vol. 31. US Government Printing Office, Washington, DC, 20 pp. US FWS and DOI Research Report 31.

[ece370759-bib-0011] Butler, P. A. 1954. “Summary of Our Knowledge of the Oyster in the Gulf of Mexico.” Fisheries Bulletin 89: 479–489.

[ece370759-bib-0012] Cake . 1983. Habitat Suitability Index Models: Gulf of Mexico American Oyster. FWS/OBS‐82/10.57. Washington DC: US Department of Interior, 37.

[ece370759-bib-0013] Calder, C. , M. Lavine , P. Muller , and J. S. Clark . 2003. “Incorporating Multiple Sources of Stochasticity Into Dynamic Population Models.” Ecology 84: 1395–1402. 10.1890/0012-9658(2003)084.

[ece370759-bib-0014] Carrier‐Belleau, C. , L. Pascal , S. D. Tiegs , C. Nozais , and P. Archambault . 2023. “Tipping Point Arises Earlier Under a Multiple‐Stressor Scenario.” Scientific Reports 13: 16780. 10.1038/s41598-023-44012-x. 37798389 PMC10555998

[ece370759-bib-0015] Casas, S. M. , D. Comba , M. K. La Peyre , S. Rikard , and J. F. La Peyre . 2023. “Rates of Osmoconformation in Triploid Eastern Oysters, and Comparison to Their Diploid Half‐Siblings.” Aquaculture 580: 740326. 10.1016/j.aquaculture.2023.740326.

[ece370759-bib-0016] Casas, S. M. , R. Lavaud , M. K. La Peyre , L. A. Comeau , R. Filgueira , and J. F. La Peyre . 2018. “Quantifying Salinity and Season Effects on Eastern Oyster Clearance and Oxygen Consumption Rates.” Marine Biology 165: 90.

[ece370759-bib-0017] Chatry, M. , R. J. Dugas , and K. A. Easley . 1983. “Optimum Salinity Regime for Oyster Production on Louisiana's State Seed Grounds.” Contributions in Marine Science 26: 81–94.

[ece370759-bib-0018] Clark, J. S. 2005. “Why Environmental Scientists Are Becoming Bayesians.” Ecology Letters 8, no. 1: 2–14.

[ece370759-bib-0019] Coen, L. D. , and A. T. Humphries . 2017. “Chapter 19: Oyster‐Generated Marine Habitats: Their Services, Enhancement, Restoration and Monitoring.” In Routledge Handbook of Ecological and Environmental Restoration, edited by S. K. Allison and S. D. Murphy . London, UK: Taylor and Francis Group.

[ece370759-bib-0020] Cote, I. M. , E. S. Darling , and C. J. Brown . 2016. “Interactions Among Ecosystem Stressors and Their Importance in Conservation.” Proceedings of the Royal Society B 283: 20152592. 10.1098/rspb.2015.2592. 26865306 PMC4760168

[ece370759-bib-0104] Coxe, N. , S. M. Casas , D. A. Marshall , M. K. La Peyre , M. W. Kelly , and J. F. La Peyre . 2023. “Differential Hypoxia Tolerance of Eastern Oysters From the Northern Gulf of Mexico at Elevated Temperature.” Journal of Experimental Marine Biology and Ecology 559: 151840. 10.1016/j.jembe.2022.151840.

[ece370759-bib-0021] CPRA, Coastal Protection and Restoration Authority . 2021. “Coastwide Reference Monitoring System (CRMS) Data.” Retrieved from Coastal Information Management System (CIMS) database. Https://cims.coastal.louisiana.gov. Accessed 12 August 2021.

[ece370759-bib-0022] Davis, H. C. , and A. Calabrese . 1964. “Combined Effects of Temperature and Salinity on Development of Eggs and Growth of Larvae of *M. Mercenaria* and *C. virginica* .” Fisheries Bulletin 64: 643–655.

[ece370759-bib-0023] de Zwaan, A. , and T. C. M. Wijsman . 1976. “Anaerobic Metabolism in Bivalvia (Mollusca): Characteristics of Anaerobic Metabolism.” Comparative Biochemistry and Physiology 54: 313–323. 6196 10.1016/0305-0491(76)90247-9

[ece370759-bib-0024] Du, J. , K. Park , C. Jensen , T. M. Dellapenna , W. G. Zhang , and Y. Shi . 2021. “Massive Oyster Kill in Galveston Bay Caused by Prolonged Low‐Salinity Exposure After Hurricane Harvey.” Science of the Total Environment 774: 145132. 10.1016/j.scitotenv.2021.145132.

[ece370759-bib-0025] Dudney, J. , and K. N. Suding . 2020. “The Elusive Search for Tipping Points.” Nature Ecology & Evolution 4: 1449–1450. 10.1038/s41559-020-1273-8. 32807946

[ece370759-bib-0106] Eierman, L. E. , and M. P. Hare . 2013. “Survival of Oyster Larvae in Different Salinities Depends on Source Population Within an Estuary.” Journal of Experimental Marine Biology and Ecology 449: 61–68. 10.1016/j.jembe.2013.08.015.

[ece370759-bib-0026] Eierman, L. E. , and M. P. Hare . 2015. “Reef‐Specific Patterns of Gene Expression Plasticity in Eastern Oysters ( *Crassostrea virginica* ).” Journal of Heredity 107: 90–100. 26245921 10.1093/jhered/esv057

[ece370759-bib-0027] Feng, Y. T. , B. J. Bethel , Y. Tian , et al. 2023. “Marine Heatwaves in the Gulf of Mexico 1983‐2021: Statistics, Recent Intensifications, and Threats on Coral Reefs.” Advances in Climate Change Research 14: 560–572. http://creativecommons.org/licenses/by‐nc‐nd/4.0/.

[ece370759-bib-0028] Ficetola, G. F. , and M. Denoël . 2009. “Ecological Thresholds: An Assessment of Methods to Identify Abrupt Changes in Species‐Habitat Relationships.” Ecography 32: 1075–1084.

[ece370759-bib-0029] Folke, C. F. , S. Carpenter , B. Wlaker , et al. 2004. “Regime Shifts, Resilience and Biodiversity in Ecosystem Management.” Annual Review of Ecology, Evolution, and Systematics 35: 557–581. https://www.jstor.org/stable/30034127.

[ece370759-bib-0030] Glandon, H. J. , A. K. Michaelis , V. A. Politano , et al. 2016. “Impact of Environment and Ontogeny on Relative Fecundity and Egg Quality of Female Oysters ( *Crassostrea virginica* ) From Four Sites in Northern Chesapeake Bay.” Biological Bulletin 231: 185–198. 28048960 10.1086/691066

[ece370759-bib-0031] Gledhill, J. H. , A. F. Barnett , M. Slattery , et al. 2020. “Mass Mortality of the Eastern Oyster *Crassostrea virginica* in the Western Mississippi Sound Following Unprecedented Mississippi River Flooding in 2019.” Journal of Shellfish Research 39: 235–244. 10.2983/035.039.0205.

[ece370759-bib-0032] GODAE, Global Ocean Data Assimilation Experiment . 2021. “Hybrid Coordinate Ocean Model (HYCOM) data.” Retrieved from Google Earth Engine database. https://code.earthengine.google.com/ee.ImageCollection. Accessed 12 October 2021.

[ece370759-bib-0033] Gosling, E. 2021. Marine Mussels: Ecology, Physiology, Genetics and Culture. England: Wiley and Sons Ltd.

[ece370759-bib-0034] Gregory, K. M. , K. McFarland , and M. P. Hare . 2023. “Reproductive Phenology of the Eastern Oyster, *Crassostrea virginica* (Gmelin, 1791), Along a Temperate Estuarine Salinity Gradient.” Estuaries and Coasts 46: 707–722.

[ece370759-bib-0035] Harley, C. D. G. , S. D. Connell , Z. A. Doubleday , et al. 2017. “Conceptualizing Ecosystem Tipping Points Within a Physiological Framework.” Ecology and Evolution 7: 6035–6045. 28808563 10.1002/ece3.3164 PMC5551099

[ece370759-bib-0036] Hayes, P. F. , and R. W. Menzel . 1981. “The Reproductive Cycle of Early Setting *Crassostrea virginica* (Gmelin) in the Northern Gulf of Mexico, and Its Implications for Population Recruitment.” Biological Bulletin 160: 80–88.

[ece370759-bib-0107] Heilmayer, O. , J. Digialleonardo , L. Qian , and G. Roesijadi . 2008. “Stress Tolerance of a Subtropical *Crassostrea virginica* Population to the Combined Effects of Temperature and Salinity.” Estuarine, Coastal and Shelf Science 79: 179–185.

[ece370759-bib-0037] Hiddink, J. G. , and M. J. Kaiser . 2005. “Implications of Liebig's Law of the Minimum for the Use of Ecological Indicators Based on Abundance.” Ecography 28: 264–271. 10.1111/j.0906-7590.2005.04063.

[ece370759-bib-0038] Hillebrand, H. , I. Donohue , W. S. Harpole , et al. 2020. “Thresholds for Ecological Responses to Global Change Do Not Emerge From Empirical Data.” Nature Ecology & Evolution 4: 1502–1509. 10.1038/s41559-020-1256-9. 32807945 PMC7614041

[ece370759-bib-0039] Hillebrand, H. , L. Kuczynski , C. Kunze , M. C. Rillo , and J. C. Dajka . 2023. “Thresholds and Tipping Points Are Tempting but Not Necessarily Suitable Concepts to Address Anthropogenic Biodiversity Change‐ An Intervention.” Marine Biodiversity 53: 43. 10.1007/s12526-023-01342-3.

[ece370759-bib-0040] Hooten, M. B. , and N. T. Hobbs . 2015. “A Guide to Bayesian Model Selection for Ecologists.” Ecological Monographs 85, no. 1: 3–28.

[ece370759-bib-0041] Isabel, L. , D. Beauchesne , C. McKindsey , and P. Archambault . 2021. “Detection of Ecological Thresholds and Selection of Indicator Taxa for Epibenthic Communities Exposed to Multiple Pressures.” Frontiers in Marine Science 8: 720710. 10.3389/fmars.2021.720710.

[ece370759-bib-0042] Jentsch, A. , J. Kreyling , and C. Beierkuhnlein . 2007. “A New Generation of Climate Change Experiments: Events, Not Trends.” Frontiers in Ecology and the Environment 5: 315–324.

[ece370759-bib-0043] Kim, C. K. , K. Park , S. P. Powers , W. M. Graham , and K. M. Bayha . 2010. “Oyster Larval Transport in Coastal Alabama: Dominance of Physical Transport Over Biological Behavior in a Shallow Estuary.” Journal of Geophysical Research 115: C10019.

[ece370759-bib-0044] La Peyre, M. K. , B. S. Eberline , T. M. Soniat , and J. F. La Peyre . 2013. “Differences in Extreme Low Salinity Timing and Duration Differentially Affect Eastern Oyster ( *Crassostrea virginica* ) Size Class Growth and Mortality in Breton Sound, LA.” Estuarine, Coastal and Shelf Science 135: 146–157. 10.1016/j.ecss.2013.10.001.

[ece370759-bib-0045] La Peyre, M. K. , B. Gossman , and J. F. La Peyre . 2009. “Defining Optimal Freshwater Flow for Oyster Production: Effects of Freshet Rate and Magnitude of Change and Duration on Eastern Oysters and *Perkinsus Marinus* Infection.” Estuaries and Coasts 32: 522–534.

[ece370759-bib-0046] La Peyre, M. K. , D. A. Marshall , and S. E. Sable . 2021. “Oyster Model Inventory: Identifying Critical Data and Modeling Approaches to Support Restoration of Oyster Reefs in Coastal U.S. Gulf of Mexico Waters.” U.S. Geological Survey Open‐File Report 2021‐10663. 10.3133/ofr20211063.

[ece370759-bib-0047] La Peyre, M. K. , A. D. Nickens , A. K. Volety , G. S. Golley , and J. F. La Peyre . 2003. “Environmental Significance of Freshets in Reducing *Perkinsus Marinus* Infection in Eastern Oysters *Crassostrea virginica* : Potential Management Applications.” Marine Ecology Progress Series 248: 165–176.

[ece370759-bib-0048] Lambert, P. C. , A. J. Sutton , P. R. Burton , K. R. Abrams , and D. R. Jones . 2005. “How Vague Is Vague? A Simulation Study of the Impact of the Use of Vague Prior Distributions in MCMC Using WinBUGS.” Statistics in Medicine 24, no. 15: 2401–2428. 10.1002/sim.2112. 16015676

[ece370759-bib-0049] Larsen, S. , and M. Alp . 2015. “Ecological Thresholds and Riparian Wetlands: An Overview for Environmental Managers.” Limnology 16: 1–9.

[ece370759-bib-0108] LDWF, Louisiana Department of Wildlife and Fisheries . 2016. “2016 Stock Assessment Report of the Public Oyster Seed Grounds and Reservations of Louisiana.” Oyster Data Report Series No. 22. https://www.wlf.louisiana.gov/assets/Resources/Publications/Stock_Assesments/Oyster/2016_Oyster_Stock_Assessment.pdf.

[ece370759-bib-0051] LDWF, Louisiana Department of Wildlife and Fisheries . 2017. “2017 Stock Assessment Report of the Public Oyster Seed Grounds and Reservations of Louisiana.” Oyster Data Report Series No. 23. Report (louisiana.gov).

[ece370759-bib-0052] LDWF, Louisiana Department of Wildlife and Fisheries . 2018a. “2018 Stock Assessment Report of the Public Oyster Seed Grounds and Reservations of Louisiana.” Oyster Data Report Series No. 24. 2018_Oyster_Stock_Assessment.pdf (louisiana.gov).

[ece370759-bib-0053] LDWF, Louisiana Department of Wildlife and Fisheries . 2018b. “LDWF Marine Fisheries Section Independent Sampling Activities Field Manual.” In 9800 Quail Drive. Baton: Rouge, LA.

[ece370759-bib-0054] LDWF, Louisiana Department of Wildlife and Fisheries . 2019. “2019 Stock Assessment Report of the Public Oyster Seed Grounds and Reservations of Louisiana.” Oyster Data Report Series No. 25. 2019‐Oyster‐Stock‐Assessment.pdf (louisiana.gov).

[ece370759-bib-0055] LDWF, Louisiana Department of Wildlife and Fisheries . 2020. “Oyster Stock Assessment of the Public Oyster Areas of Louisiana.” Oyster Data Report Series No. 26.2020‐Oyster‐Stock‐Assessment.pdf (louisiana.gov).

[ece370759-bib-0056] Leonhardt, J. M. , S. Casas , J. E. Supan , and L. Peyre . 2017. “Stock Assessment for Eastern Oyster Seed Production and Field Grow‐Out in Louisiana.” Aquaculture 466: 9–19.

[ece370759-bib-0057] Lindquist, D. C. , S. E. Sable , L. D'Acunto , et al. 2021. “Habitat Suitability Index Model Improvements.” In Master Plan: Attachment C10: 2023 Habitat Suitability Index (HSI) Model, vol. Version 3, 1–35. Baton Rouge, LO: Coastal Protection and Restoration Authority.

[ece370759-bib-0058] Livingston, R. J. , F. G. Lewis , G. C. Woodsum , et al. 2000. “Modeling Oyster Population Response to Variation in Freshwater Input.” Estuarine, Coastal and Shelf Science 50: 655–672.

[ece370759-bib-0059] Loosanoff, V. L. 1952. “Behavior of Oysters in Water of Low Salinity.” Proceedings of the National Shellfisheries Association 43: 135–151.

[ece370759-bib-0060] Lowe, M. R. , T. Sehlinger , T. M. Soniat , and M. K. La Peyre . 2017. “Interactive Effects of Water Temperature and Salinity on Growth and Mortality of Eastern Oysters, *Crassostrea virginica* : A Meta‐Analysis Using 40 Years of Monitoring Data.” Journal of Shellfish Research 36: 1–15.

[ece370759-bib-0061] Luo, M. , S. Wu , G. Ngar‐Cheung Lau , et al. 2024. “Anthropogenic Forcing Has Increased the Risk of Longer‐Traveling and Slower‐Moving Large Contiguous Heatwaves.” Science Advances 10: eadl1598. 10.1126/sciadv.adl1598. 38552023 PMC10980275

[ece370759-bib-0062] Marshall, D. A. , S. M. Casas , W. C. Walton , et al. 2021. “Divergence in Salinity Tolerance of Northern Gulf of Mexico Eastern Oysters Under Field and Laboratory Exposure.” 10.1093/conphys/coab065. PMC8384081 34447578

[ece370759-bib-0063] Marshall, D. A. , N. C. Coxe , M. K. La Peyre , et al. 2021. “Tolerance of Northern Gulf of Mexico Eastern Oysters to Chronic Warming at Extreme Salinities.” Journal of Thermal Biology 100: 103072. 34503809 10.1016/j.jtherbio.2021.103072

[ece370759-bib-0064] Marshall, D. A. , S. C. Moore , M. Sutor , and J. F. La Peyre . 2020. “La Peyre MK.2020. Using Reproductive Potential to Assess Oyster Population Sustainability.” Restoration Ecology 28: 1621–1632. 10.1111/rec.13225.

[ece370759-bib-0065] McFarland, K. , D. Rumbold , A. N. Loh , et al. 2022. “Effects of Freshwater Release on Oyster Reef Density, Reproduction, and Disease in a Highly Modified Estuary.” Environmental Monitoring and Assessment 194: 96. 10.1007/s10661-021-09489-x. 35029759

[ece370759-bib-0103] McFarland, K. , J. Vignier , E. Standen , and A. K. Volety . 2022. “Synergistic Effects of Salinity and Temperature on the Eastern Oyster *Crassostrea virginica* Throughout the Lifespan.” Marine Ecology Progress Series 700: 111–124.

[ece370759-bib-0066] McFarland, K. , J. Vignier , E. Standen , and A. K. Volety . 2022. “Synergistic Effects of Salinity and Temperature on the Eastern Oyster *Crassostrea virginica* Throughout the Lifespan.” Marine Ecology Progress Series 700: 111–124. 10.3354/meps14178.

[ece370759-bib-0067] Morris, R. L. , M. K. La Peyre , B. M. Webb , et al. 2021. “Large‐Scale Variation in Wave Attenuation of Oyster Reef Living Shorelines and the Influence of Inundation Duration.” Ecological Applications 31: 1–15. 10.1002/Eap.2382. 34042243

[ece370759-bib-0068] Mroch, R. M., III , D. B. Eggleston , and B. J. Puckett . 2012. “Spatiotemporal Variation in Oyster Fecundity and Reproductive Output in a Network of No‐Take Reserves.” Journal of Shellfish Research 31: 1091–1101.

[ece370759-bib-0069] Munroe, D. , A. Tabatabai , I. Burt , D. Bushek , E. N. Powell , and J. Wilkin . 2013. “Oyster Mortality in Delaware Bay: Impacts and Recovery From Hurricane Irene and Tropical Storm Lee.” Estuarine, Coastal and Shelf Science 135: 209–219.

[ece370759-bib-0070] Myhre, G. , K. Alterskjaer , C. W. Stjern , et al. 2019. “Frequency of Extreme Precipitation Increases Extensively With Even Rareness Under Global Warming.” Scientific Reports 9: 16063. 10.1038/s41598-019-52277-4. 31690736 PMC6831572

[ece370759-bib-0071] NLCD . 2016. “Multi‐Resolution Land Characteristics Consortium (U.S.).” In National Land Cover Dataset (NLCD), Vol. 2016. Multi‐Resolution Land Characteristics Consortium, Research Triangle Park, NC; in Dewitz J. 2019. National Land Cover Database (NLCD) 2016 Products (Ver.3.0, November 2023): U.S. Geological Survey Data Release. 10.5066/P96HHBIE.

[ece370759-bib-0073] NOAA, National Oceanic and Atmospheric Administration . 2022. Heat Stress Datasets and Documentation. Accessed 11 November 2023. https://www.epa.gov/climate‐indicators/climate‐change‐indicators‐heat‐waves#ref7.

[ece370759-bib-0074] North, E. W. , Z. Schlag , R. R. Hood , et al. 2008. “Vertical Swimming Behavior Influences the Dispersal of Simulated Oyster Larvae in a Coupled Particle‐Tracking and Hydrodynamic Model of Chesapeake Bay.” Marine Ecology Progress Series 359: 99–115.

[ece370759-bib-0075] Peck, L. S. , M. S. Clark , S. A. Morley , A. Massey , and H. Rossetti . 2009. “Animal Temperature Limits and Ecological Relevance: Effects of Size, Activity and Rates of Change.” Functional Ecology 23: 248–256.

[ece370759-bib-0076] Plummer, M. 2003. “JAGS: A Program for Analysis of Bayesian Graphical Models Using Gibbs Sampling. Proceedings of the 3rd International Workshop on Distributed Statistical Computing. 124:125.10:1‐10.” http://mcmc‐jags.sourceforge.net.

[ece370759-bib-0077] Plummer, M. 2024. “_rjags: Bayesian Graphical Models Using MCMC_. R Package Version 4‐16.” https://CRAN.R‐project.org/package=rjags.

[ece370759-bib-0078] Prein, A. F. , C. Liu , K. Ikeda , et al. 2017. “Increased Rainfall Volume From Future Convective Storms in the US.” Nature Climate Change 7: 880–884. https://www.nature.com/articles/s41558‐017‐0007‐7.

[ece370759-bib-0079] Probst, P. , M. Wright , and C. Boulesteix . 2018. “Hyperparameters and Tuning Strategies for Random Forest.” WIREs Data Mining and Knowledge Discovery. 10.1002/widm.1301.

[ece370759-bib-0080] Pruett, J. L. , A. F. Pandelides , K. L. Willett , and D. J. Gochfeld . 2021. “Effects of Flood‐Associated Stressors on Growth and Survival of Early Life Stage Oysters ( *Crassostrea virginica* ).” Journal of Experimental Marine Biology and Ecology 544: 151615. 10.1016/j.jembe.2021.151615.

[ece370759-bib-0081] Rilov, G. , A. D. Mazaris , V. Stelzenmuller , and B. Helmuth . 2019. “Adaptive Marine Conservation Planning in the Face of Climate Change: What Can We Learn From Physiological, Genetic and Ecological Studies?” Global Ecology and Conservation 17: e00566.

[ece370759-bib-0082] Rostami, M. A. , F. Frontalini , P. Giordano , et al. 2021. “Testing the Applicability of Random Forest Modeling to Examine Benthic Foraminiferal Responses to Multiple Environmental Parameters.” Marine Environmental Research 172: 105502. 34638002 10.1016/j.marenvres.2021.105502

[ece370759-bib-0083] Roubeix, V. , P. A. Danis , T. Feret , and J. M. Baudoin . 2016. “Identification of Ecological Thresholds From Variations in Phytoplankton Communities Among Lakes: Contribution to the Definition of Environmental Standards.” Environmental Monitoring and Assessment 188: 246. 10.1007/s10661-016-5238-y. 27010711

[ece370759-bib-0084] Rybovich, M. , M. K. La Peyre , S. G. Hall , and J. F. La Peyre . 2016. “Increased Temperatures Combined With Lowered Salinities Differentially Impact Oyster Size Class Growth and Mortality.” Journal of Shellfish Research 35: 101–113.

[ece370759-bib-0085] Scheffer, M. , J. Bascompte , W. A. Brock , et al. 2009. “Early‐Warning Signals for Critical Transitions.” Nature 461: 53–59. 19727193 10.1038/nature08227

[ece370759-bib-0086] Scheffer, M. , S. Carpenter , J. A. Foley , C. Folke , and B. Walker . 2001. “Catastrophic Shifts in Ecosystems.” Nature 413: 591–596. 11595939 10.1038/35098000

[ece370759-bib-0087] Schielzeth, H. , and S. Nakagawa . 2013. “Nested by Design: Model Fitting and Interpretation in a Mixed Model Era.” Methos in Ecology and Evolution 4, no. 1: 14–24. 10.1111/j.2041-210x2012.00251.x.

[ece370759-bib-0088] Sehlinger, T. , M. R. Lowe , M. K. La Peyre , and T. M. Soniat . 2019. “Differential Effects of Temperature and Salinity on Growth and Mortality of Oysters ( *Crassostrea virginica* ) in Barataria Bay and Breton Sound, Louisiana.” Journal of Shellfish Research 38: 317–326.

[ece370759-bib-0089] Shelford, V. E. 1931. “Some Concepts of Bioecology.” Ecology 12: 455–467. 10.2307/1928991.

[ece370759-bib-0090] Shumway, S. E. 1996. “Natural Environmental Factors.” In The Eastern Oyster *Crassostrea virginica* , edited by V. S. Kennedy , R. I. E. Newell , and A. F. Eble , 467–513. Maryland Sea Grant College: College Park, MD.

[ece370759-bib-0091] Sirovy, K. A. , S. M. Casas , J. F. La Peyre , and M. W. Kelly . 2023. “Population‐Specific Responses in Eastern Oysters Exposed to Low Salinity in the Northern Gulf of Mexico.” Experimental Biology 226, no. 14: jeb244315. 10.1242/jeb.244315. 37350275

[ece370759-bib-0092] Sokolova, I. M. , M. Frederich , R. Bagwe , G. Lanning , and A. A. Sukhotin . 2012. “Energy Homeostasis as an Integrative Tool for Assessing Limits of Environmental Stress Tolerance in Aquatic Invertebrates.” Marine Environmental Research 79: 1–15. 22622075 10.1016/j.marenvres.2012.04.003

[ece370759-bib-0105] Soniat, T. M. , and M. S. Brody . 1988. “Field Validation of a Habitat Suitability Index Model for the American Oyster.” Estuaries 11, no. 2: 87–95.

[ece370759-bib-0093] Soniat, T. M. , J. M. Klinck , E. N. Powell , et al. 2012. “A Shell‐Neutral Modeling Approach Yields Sustainable Oyster Harvest Estimates: A Retrospective Analysis of the Louisiana State Primary Seed Grounds.” Journal of Shellfish Research 4: 1103–1112.

[ece370759-bib-0094] Southworth, M. , M. C. Long , and R. Mann . 2017. “Oyster ( *Crassostrea virginica* [Gmelin, 1791]) Mortality at Prolonged Exposures to High Temperature and Low Salinity.” Journal of Shellfish Research 36: 335–340.

[ece370759-bib-0095] Swam, L. M. , B. Couvillion , B. Callam , J. F. La Peyre , and M. K. La Peyre . 2022. “Defining Oyster Resource Zones Across Coastal Louisiana for Restoration and Aquaculture.” Ocean and Coastal Management 225: 106178. 10.1016/j.ocecoaman.2022.106178.

[ece370759-bib-0096] Tang, J. , and W. J. Riley . 2021. “Finding Liebig's Law of the Minimum.” Ecological Applications 31: e02458. 10.1002/eap.2458. 34529311 PMC9285345

[ece370759-bib-0097] Toms, J. D. , and M. A. Villard . 2015. “Threshold Detection: Matching Statistical Methodology to Ecological Questions and Conservation Planning Objectives.” Avian Conservation and Ecology 10, no. 1: 2.

[ece370759-bib-0098] Truebano, M. , P. Fenner , O. Tills , S. D. Rundle , and E. L. Rezende . 2018. “Thermal Strategies Vary With Life History Stage.” Journal of Experimental Biology 221, no. 8: jeb171629. 10.1242/jeb.171629. 29559547

[ece370759-bib-0099] USGS, United States Geological Survey . 2021. “USGS Current Condition for the Nation.” Accessed 23 August 2021. National Water Information System: Web Interface.

[ece370759-bib-0100] Wu, W. , M. Bethel , D. R. Mishra , and T. Hardy . 2018. “Model Selection in Bayesian Framework to Identify the Best WorldView‐2 Based Vegetation Index in Predicting Green Biomass of Salt Marshes in the Northern Gulf of Mexico.” GIScience & Remote Sensing 55: 880–904. 10.1080/15481603.2018.1460934.

[ece370759-bib-0101] Wu, W. , P. D. Biber , M. S. Peterson , and C. Gong . 2012. “Modeling Photosynthesis of *Spartina alterniflora* (Smooth Cordgrass) Impacted by the Deepwater Horizon Oil Spill Using Bayesian Inference.” Environmental Research Letters 7: 045302.

[ece370759-bib-0102] Zabin, C. J. , L. J. Jurgens , J. M. Bible , et al. 2022. “Increasing the Resilience of Ecological Restoration to Extreme Climatic Events.” Frontiers in Ecology and the Environment 20: 310–318. 10.1002/fee.2471.

