# Supplementary material for: Multiple Dimensions Define Thresholds for Population Resilience of the Eastern Oyster, Crassostrea virginica
Source: Ecol Evol. 2025 Jan 21;15(1):e70759. doi: 10.1002/ece3.70759 (PMC11747143; doi:10.1002/ece3.70759)
Supplement: Supplementary file 1 — Data S1. [file ECE3-15-e70759-s001.docx]

**Multiple dimensions define thresholds for population resilience of the eastern oyster, *Crassostrea virginica***

**Submitted to: Ecology and Evolution**

Revision 1: October 14, 2024

Supplementary Files: 2 Supplementary Tables; 4 Supplementary Figures; 2 Supplementary Code

Megan K. La Peyre^1,2*^, Hongqing Wang^3^, Shaye Sable^4^, Wei Wu^5^, Bin Li^6^, Devin Comba^2^, Carlos Perez^7^, Melanie Bates^2^, Lauren Swam^2^

1 U.S. Geological Survey, Louisiana Cooperative Fish and Wildlife Research Unit, Baton Rouge, LA

2 School of Renewable Natural Resources, Louisiana State University Agricultural Center, Baton Rouge, LA

3 U.S. Geological Survey, Wetland and Aquatic Research Center, Baton Rouge, LA

4 Dynamic Solutions, LLC, Baton Rouge, LA

5 Division of Coastal Sciences, School of Ocean Science and Engineering, The University of Southern Mississippi, Ocean Springs, MS

6 Department of Experimental Statistics, Louisiana State University, Baton Rouge, LA

7 GEC Inc, Baton Rouge, LA

*Contact : Megan La Peyre, [mlapeyre@agcenter.lsu.edu](mailto:mlapeyre@agcenter.lsu.edu)

**Supplementary Tables**

Supplementary Table S1. Multi-level Bayesian model comparison to determine the optimum levels in the models used to define thresholds for population resilience of the eastern oyster, *Crassostrea virginica*, based on lowest deviance information criteria (DIC) and posterior predictive loss (PPL). Models were run by oyster life stage (spat: < 25 mm shell height; adult: ≥ 25 mm shell height), season (fall: September-October; Winter: November-December) and region (east; west). Spatial and temporal levels reflect model levels of sample station (S), estuarine basin (B, Figure 1), and year (Y; 2016-2020), and all models included days of all salinity levels, as total number of summer days (total) and maximum continuous number of days (Maximum) of salinity <1, salinity of 1-3, salinity of 3-5, salinity 5-7, and salinity of 7-9). Selected models are indicated in bold.

| Response variables | | Region | Spatial/temporal Levels | Salinity Count | DIC | PPL |
| --- | --- | --- | --- | --- | --- | --- |
| Life stage | Season |  |  |  |  |  |
| Spat | Fall | East | **Station nested within basin + Year** | **Maximum** | **3928.08** | **311076.3** |
|  |  |  | Station nested within basin | Maximum | 3928.42 | 312364.3 |
|  |  |  | Basin + Year | Maximum | 4124.72 | 581670.3 |
|  |  |  | Basin | Maximum | 4125.10 | 584391.9 |
|  |  |  | Year | Maximum | 4155.06 | 622520.4 |
|  |  |  | None | Maximum | 4156.08 | 629593.8 |
|  |  |  | Station nested within basin + Year | Total | 3933.95 | 316094.5 |
|  |  |  | Station nested within basin | Total | 3935.47 | 318644.5 |
|  |  |  | Basin + Year | Total | 4110.27 | 563552.2 |
|  |  |  | Basin | Total | 4110.07 | 564289.9 |
|  |  |  | Year | Total | 4149.96 | 612871.4 |
|  |  |  | None | Total | 4143.01 | 611716.7 |
|  |  | West | Station nested within basin + Year | Maximum | 1000.90 | 52022.0 |
|  |  |  | Station nested within basin | Maximum | 1026.17 | 67121.3 |
|  |  |  | Basin + Year | Maximum | 1040.25 | 84113.6 |
|  |  |  | Basin | Maximum | 1056.34 | 98950.4 |
|  |  |  | Year | Maximum | 1040.14 | 84463.1 |
|  |  |  | None | Maximum | 1053.14 | 98155.2 |
|  |  |  | **Station nested within basin + Year** | **Total** | **997.34** | **51162.9** |
|  |  |  | Station nested within basin | Total | 1026.62 | 67306.3 |
|  |  |  | Basin + Year | Total | 1034.34 | 80121.1 |
|  |  |  | Basin | Total | 1052.30 | 95522.8 |
|  |  |  | Year | Total | 1032.93 | 79935.4 |
|  |  |  | None | Total | 1048.14 | 93657.5 |
| Spat | Winter | East | **Station nested within basin + Year** | **Maximum** | **3565.12** | **324985.0** |
|  |  |  | Station nested within basin | Maximum | 3581.12 | 330767.4 |
|  |  |  | Basin + Year | Maximum | 3708.15 | 438004.2 |
|  |  |  | Basin | Maximum | 3711.04 | 441488.3 |
|  |  |  | Year | Maximum | 3761.14 | 475817.4 |
|  |  |  | None | Maximum | 3762.02 | 475585.4 |
|  |  |  | Station nested within basin + Year | Total | 3568.93 | 328662.2 |
|  |  |  | Station nested within basin | Total | 3582.48 | 334660.2 |
|  |  |  | Basin + Year | Total | 3691.48 | 428596.5 |
|  |  |  | Basin | Total | 3693.92 | 430555.5 |
|  |  |  | Year | Total | 3737.93 | 463994.7 |
|  |  |  | None | Total | 3740.02 | 463812.0 |
|  |  | West | Station nested within basin + Year | Maximum | 927.72 | 62823.5 |
|  |  |  | Station nested within basin | Maximum | 941.16 | 69724.0 |
|  |  |  | Basin + Year | Maximum | 959.49 | 78396.1 |
|  |  |  | Basin | Maximum | 963.63 | 83572.8 |
|  |  |  | Year | Maximum | 959.70 | 77441.3 |
|  |  |  | None | Maximum | 962.76 | 82719.0 |
|  |  |  | **Station nested within basin + Year** | **Total** | **925.91** | **62691.0** |
|  |  |  | Station nested within basin | Total | 937.42 | 69542.0 |
|  |  |  | Basin + Year | Total | 956.61 | 77254.8 |
|  |  |  | Basin | Total | 959.40 | 82020.4 |
|  |  |  | Year | Total | 955.73 | 75313.7 |
|  |  |  | None | Total | 957.02 | 80641.1 |
| Adult | Fall | East | **Station nested within basin + Year** | **Maximum** | **3934.28** | **349837.1** |
|  |  |  | Station nested within basin | Maximum | 3937.34 | 351772.6 |
|  |  |  | Basin + Year | Maximum | 4240.49 | 682468.7 |
|  |  |  | Basin | Maximum | 4243.99 | 688502.7 |
|  |  |  | Year | Maximum | 4264.04 | 723433.5 |
|  |  |  | None | Maximum | 4270.00 | 732142.9 |
|  |  |  | Station nested within basin + Year | Total | 3934.52 | 354187.0 |
|  |  |  | Station nested within basin | Total | 3941.80 | 357406.9 |
|  |  |  | Basin + Year | Total | 4210.84 | 653165.1 |
|  |  |  | Basin | Total | 4212.10 | 656242.4 |
|  |  |  | Year | Total | 4231.93 | 698613.5 |
|  |  |  | None | Total | 4233.04 | 695250.3 |
|  |  | West | **Station nested within basin + Year** | **Maximum** | **1080.26** | **122596.8** |
|  |  |  | Station nested within basin | Maximum | 1080.50 | 122774.8 |
|  |  |  | Basin + Year | Maximum | 1137.78 | 169246.5 |
|  |  |  | Basin | Maximum | 1137.10 | 169665.1 |
|  |  |  | Year | Maximum | 1140.26 | 163589.7 |
|  |  |  | None | Maximum | 1135.12 | 165382.6 |
|  |  |  | Station nested within basin + Year | Total | 1079.60 | 122673.3 |
|  |  |  | Station nested within basin | Total | 1078.34 | 123257.0 |
|  |  |  | Basin + Year | Total | 1133.91 | 166167.6 |
|  |  |  | Basin | Total | 1134.05 | 166790.6 |
|  |  |  | Year | Total | 1140.27 | 161709.1 |
|  |  |  | None | Total | 1134.06 | 164545.7 |
| Adult | Winter | East | **Station nested within basin + Year** | **Maximum** | **3730.11** | **300716.1** |
|  |  |  | Station nested within basin | Maximum | 3737.13 | 302537.8 |
|  |  |  | Basin + Year | Maximum | 3997.61 | 523879.4 |
|  |  |  | Basin | Maximum | 4002.05 | 527827.6 |
|  |  |  | Year | Maximum | 4008.06 | 544423.4 |
|  |  |  | None | Maximum | 4014.00 | 549188.6 |
|  |  |  | Station nested within basin + Year | Total | 3737.61 | 306270.8 |
|  |  |  | Station nested within basin | Total | 3744.99 | 308366.9 |
|  |  |  | Basin + Year | Total | 3979.28 | 509787.9 |
|  |  |  | Basin | Total | 3980.40 | 511211.1 |
|  |  |  | Year | Total | 3988.12 | 531515.4 |
|  |  |  | None | Total | 3987.99 | 527413.8 |
|  |  | West | Station nested within basin + Year | Maximum | 1087.75 | 108892.6 |
|  |  |  | **Station nested within basin** | **Maximum** | **1086.42** | **108532.8** |
|  |  |  | Basin + Year | Maximum | 1118.54 | 147977.1 |
|  |  |  | Basin | Maximum | 1118.18 | 147870.0 |
|  |  |  | Year | Maximum | 1122.21 | 146846.1 |
|  |  |  | None | Maximum | 1114.17 | 145160.9 |
|  |  |  | Station nested within basin + Year | Total | 1089.35 | 110640.2 |
|  |  |  | Station nested within basin | Total | 1088.98 | 110060.9 |
|  |  |  | Basin + Year | Total | 1117.68 | 146312.9 |
|  |  |  | Basin | Total | 1117.13 | 146071.8 |
|  |  |  | Year | Total | 1120.32 | 145650.9 |
|  |  |  | None | Total | 1113.14 | 143690.2 |

Supplementary Table S2: Identification of the most informative covariate, relationship, and probability using the selected models identified in Supplementary Table S1 for the multi-level Bayesian analysis. The models with only one covariate and the smallest and/or second smallest deviance information criteria (DIC) are selected for each response variable, indicated in bold. If the DICs’ difference is within 2, the models are considered similar. These selected outcomes are summarized in Tables 3-4 in the main manuscript. Models were run by oyster life stage (Table 2A: spat: < 25 mm shell height; Table 2B: adult: ≥ 25 mm shell height), season (fall: September-October; Winter: November-December) and region (east; west). Spatial and temporal levels indicated in “Optimum levels” reflect model levels of sample station (S), estuarine basin (B, Figure 1), and year (Y; 2016-2020), and are identified in Supplementary Table S1; ‘S nest B +Y’ indicates ‘station nested in basin and year’. Salinity Count (SC) is either total number of days of summer salinity below a certain threshold (T), or maximum continuous number of days within an identified salinity threshold (M). Results indicate the salinity level, whether the relationship identified is negative (-) or positive (+), while probability is the probability that either 95% of credible interval (CI) of coefficient for the selected covariate does not overlap 0, or 50% of probability indicates that 50% of CI does not overlap 0 while 95% of CI does, and “/” indicates that both 50% and 95% of CIs overlap 0. Models selected are highlighted in bold and grey boxes.

Supplementary Table S2A

| Response variable | | | Optimum | Results | | | | | |
| --- | --- | --- | --- | --- | --- | --- | --- | --- | --- |
| Life stage | Season | Region |  | DIC | Salinity Count | Salinity | +/- | CI (%) | Quadratic |
| **Spat** | Fall | East | S nest B + Y | 3928.08 | M | < 1 | - | 95 |  |
|  |  |  |  |  |  | 3-5 | - | 50 |  |
|  |  |  |  |  |  | 5-7 | + | 50 |  |
|  |  |  |  | 3925.58 |  | <1 | - | 95 |  |
|  |  |  |  | 3924.56 |  | <3 | - | 95 |  |
|  |  |  |  | **3922.06** |  | **<5** | **-** | **95** |  |
|  |  | West | S nest B + Y | 994.28 | T | < 1 | + | 50 |  |
|  |  |  |  |  |  | <9 | + | 95 |  |
|  |  |  |  | 998.60 |  | < 1 | + | 50 |  |
|  |  |  |  | **992.57** |  | **< 9** | **+** | **95** |  |
|  |  |  |  | 997.88 |  | < 3 | / | / |  |
|  |  |  |  | 999.78 |  | <5 | + | 50 |  |
|  |  |  |  | 993.65 |  | < 9 | + | 50 | 7-9 (- 50%) |
|  |  |  |  | 998.38 |  | 3-5 | + | 50 |  |
|  |  |  |  | **995.04** |  | **1-3** | **-** | **50** |  |
|  | Winter | East | S nest B + Y | **3560.37** | M | **5-7** | **+** | **95** |  |
|  |  |  |  | 3565.39 |  | < 1 | / | / |  |
|  |  |  |  | 3566.04 |  | < 3 | / | / |  |
|  |  |  |  | 3566.4 |  | < 5 | / | / |  |
|  |  |  |  | 3565.97 |  | 1-3 | + | 50 |  |
|  |  |  |  | 3564.67 |  | 3-5 | / | / |  |
|  |  |  |  | 3559.53 |  | 5-7 | + | 95 | 5-7 (-, 95%) |
|  |  | West | S nest B + Y | 920.55 | T | 1-3 | - | 50 |  |
|  |  |  |  |  |  | 3-5 | + | 50 |  |
|  |  |  |  | **916.56** |  | **1-3** | **-** | **50** |  |
|  |  |  |  | 923.09 |  | 3-5 | + | 50 |  |
|  |  |  |  | 922.68 |  | < 1 | / | / |  |
|  |  |  |  | 920.76 |  | <3 | / | / |  |
|  |  |  |  | 922.68 |  | < 5 | / | / |  |

Supplementary Table S2B

| Response variable | | | Optimum | Results | | | | | |
| --- | --- | --- | --- | --- | --- | --- | --- | --- | --- |
| Life stage | Season | Region |  | DIC | Salinity Count | Salinity | +/- | CI (%) | Quadratic |
| **Adult** | Fall | East | S nest B + Y | **3931.02** | **M** | **< 1** | **-** | **95** |  |
|  |  | West | S nest B+Y | **1071.31** | **M** | **5-7** | **/** | **/** |  |
|  |  |  |  | **1072.18** |  | **< 1** | **/** | **/** |  |
|  |  |  |  | 1072.27 |  | < 3 | / | / |  |
|  |  |  |  | 1073.17 |  | < 5 | / | / |  |
|  |  |  |  | 1074.37 |  | 5-7 | + | 50 | 5-7 |
|  | Winter | East | S nest B + Y | 3727.99 | M | < 1 | + | 50 |  |
|  |  |  |  |  |  | 1-3 | + | 50 |  |
|  |  |  |  |  |  | 3-5 | - | 50 |  |
|  |  |  |  |  |  | < 9 | + | 50 |  |
|  |  |  |  | **3725.55** |  | **< 9** | **+** | **50** |  |
|  |  |  |  | 3728.81 |  | < 9 | + | 95 |  |
|  |  |  |  | 3726.00 |  | < 1 | - | 50 |  |
|  |  |  |  | 3726.97 |  | 1-3 | + | 50 |  |
|  |  |  |  | 3727.22 |  | < 3 | / | / |  |
|  |  |  |  | 3726.53 |  | 3-5 | / | / |  |
|  |  |  |  | 3726.22 |  | < 5 | / | / |  |
|  |  | West | S nest B | 1079.33 | M | < 1 | + | 50 |  |
|  |  |  |  | 1079.65 |  | < 3 | / | / |  |
|  |  |  |  | 1080.56 |  | < 3 | / | / |  |
|  |  |  |  | **1078.58** |  | **3-5** | **/** | **/** |  |
|  |  |  |  | 1080.37 |  | < 5 | / | / |  |
|  |  |  |  | 1079.58 |  | < 7 | / | / |  |
|  |  |  |  | 1079.7 |  | < 9 | / | / |  |
|  |  |  |  | 1079.54 |  | 7-9 | / | / |  |
|  |  |  |  | 1079.35 |  | 5-7 | / | / |  |
|  |  |  |  | 1080.28 |  | < 1 | / | / | <1 |

**Supplementary Figures**

**Supplementary Figure S1** Louisiana oyster growing estuarine areas, indicating existing mapped oyster reef areas, designated as cultch plants (shell reefs), reef bottoms, and public seed grounds where oysters are harvested, and, within each year map, (A) maximum continuous number of days of salinity below the identified threshold (Salinity < 1, < 3, < 5) during a calendar year, (B) maximum continuous number of days of salinity below the identified threshold (Salinity < 1, < 3, < 5) during the summer (May 1-August 31), (C) total number of days of salinity below the identified threshold (Salinity < 1, < 3, < 5) during a calendar year, and (D) total number of days of salinity below the identified threshold of salinity < 1 (MAPS_1), salinity < 3 (MAPS_3), and salinity < 5 (MAPS_%) during the summer (May 1-August 31). Daily salinity data were from Swam et al. (2023). Map cover is from the National Land Cover Dataset (NLCD 2016).

**MAPS_1**


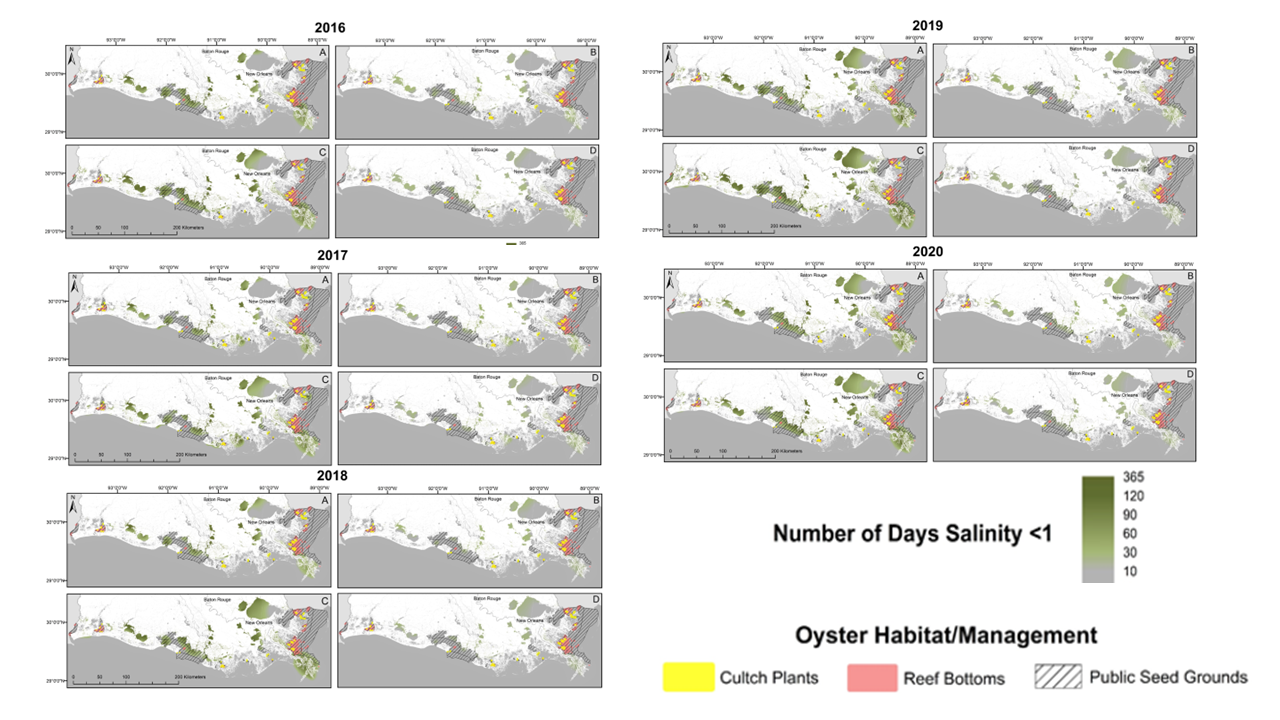


**MAPS_3**


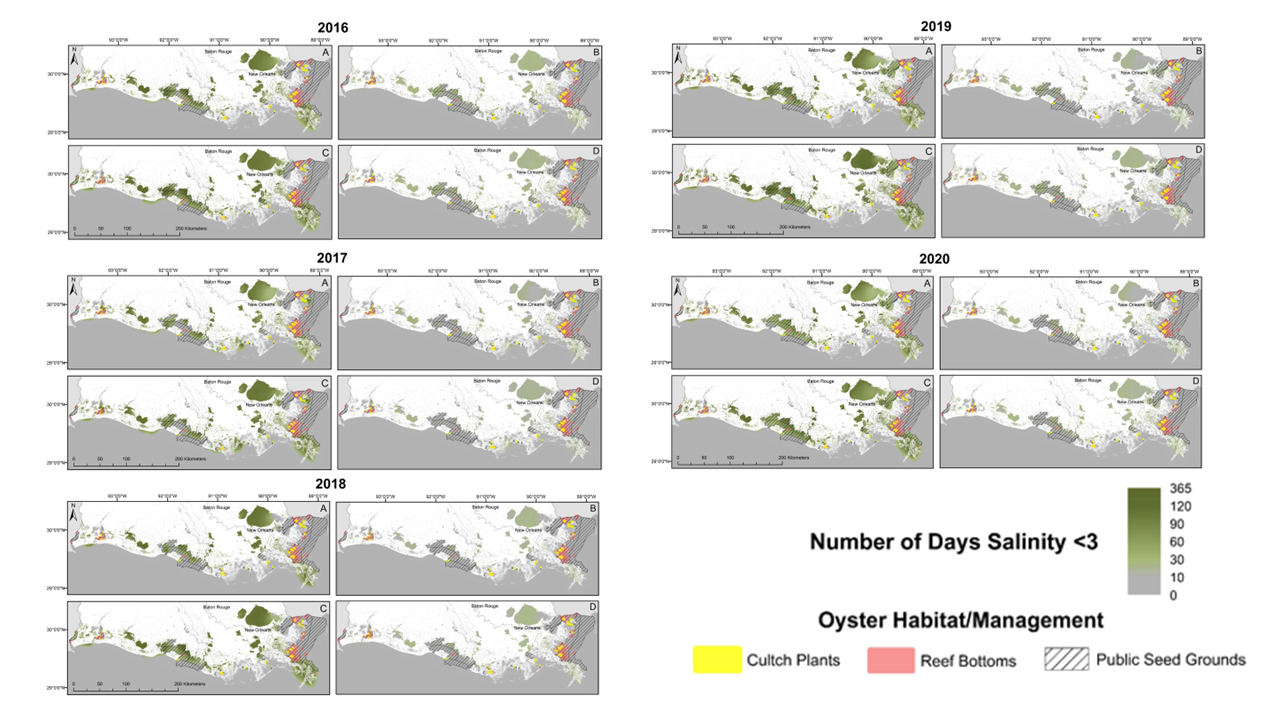


**MAPS_5**


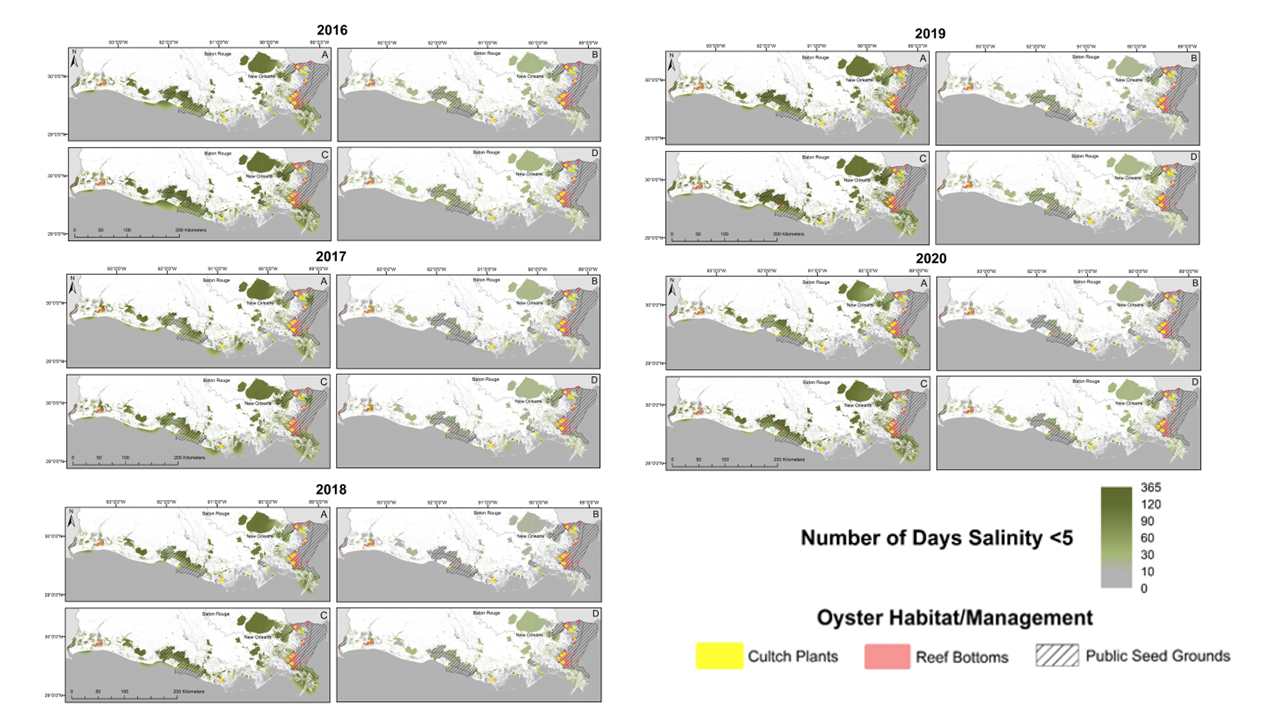


**Supplementary Figure S2.** Provided below are all the equations corresponding to the full model in Figure 2. The symbols are described in the caption of Figure 2: $A_{ijk}$denotes abundance at Station j in Basin i in Year k. $S_{<1}, S_{1-3}, S_{3-5}, S_{5-7}, S_{7-9}$ denote annual or summer total number of days (or maximum of consecutive days) of salinity less than 1, 1-3, 3-5, 5-7, and 7-9 respectively, and γ_1-5_ denote the coefficients associated with them. $\beta_{ij,0}$denotes the intercept contributed by a station scale, which is nested within a basin and sampled from a parameter at the basin scale $\alpha_{i\cdot,0}$. λ_k,0_ denotes the intercept contributed by the annual scale. $\sigma^{2}$, $\sigma_{j}^{2}$, $\sigma_{i}^{2},\sigma_{k}^{2}$ denote variances at the oyster reef, station, basin, and annual scales.


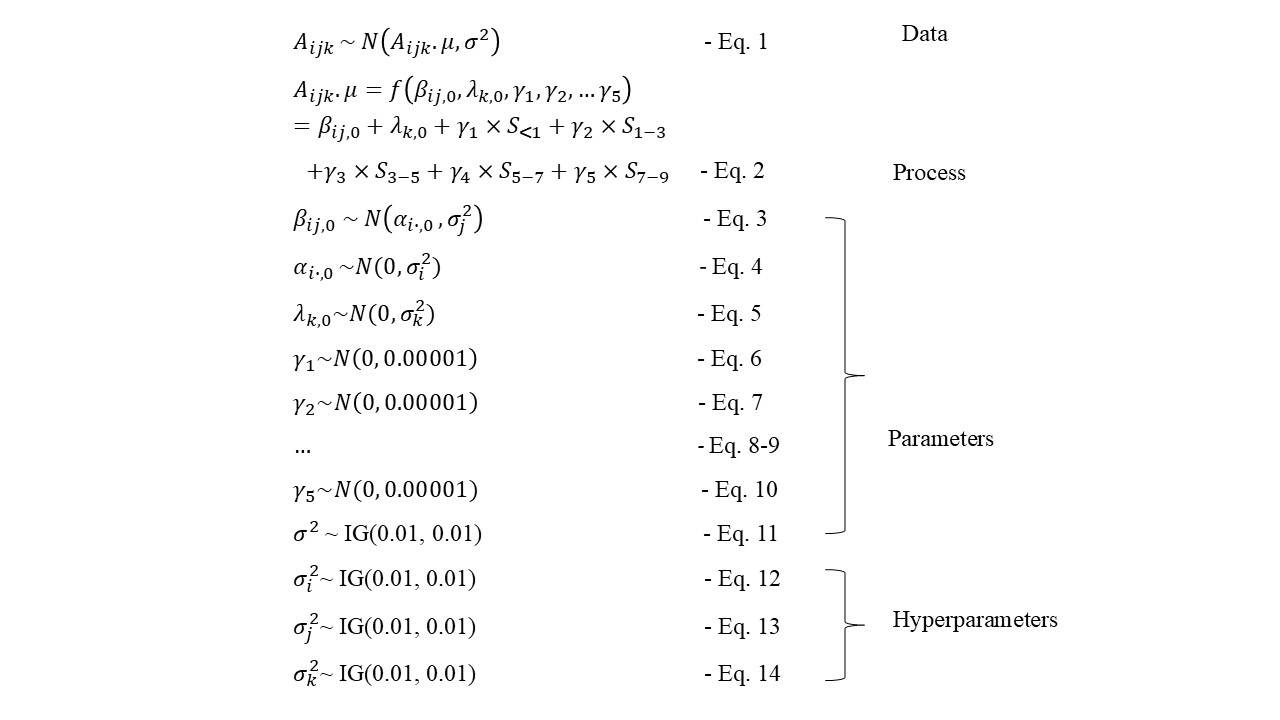


**Supplementary Figure S3.** Variable importance and partial dependence plot of variables identified for interpretation as critical lower salinity threshold spat (< 25 mm shell height) or adult (≥ 25 mm shell height) abundance during fall (September/October) or winter (November/December) monitoring completed by Louisiana Department of Wildlife and Fisheries during their long-term oyster monitoring collection for Louisiana East Basins (Pontchartrain + Barataria + Terrebonne basins). Variable used for importance was either the variable of highest importance, or, when the top three variables were clustered in importance, the lowest salinity range of the top three variables. Variables explored included the total number (s) or the maximum continuous number (c) of days where salinity was within summer (su) salinity bins of < 1, 1 - <3, 3 < 5, 5 < 7, 7 < 9, and are coded such that the variable for total number of days of salinity between within the 7 < 9 bin is s_d_su7.9, or maximum continuous number of days within the 7 < 9 bin is c.d_su7.9.

**
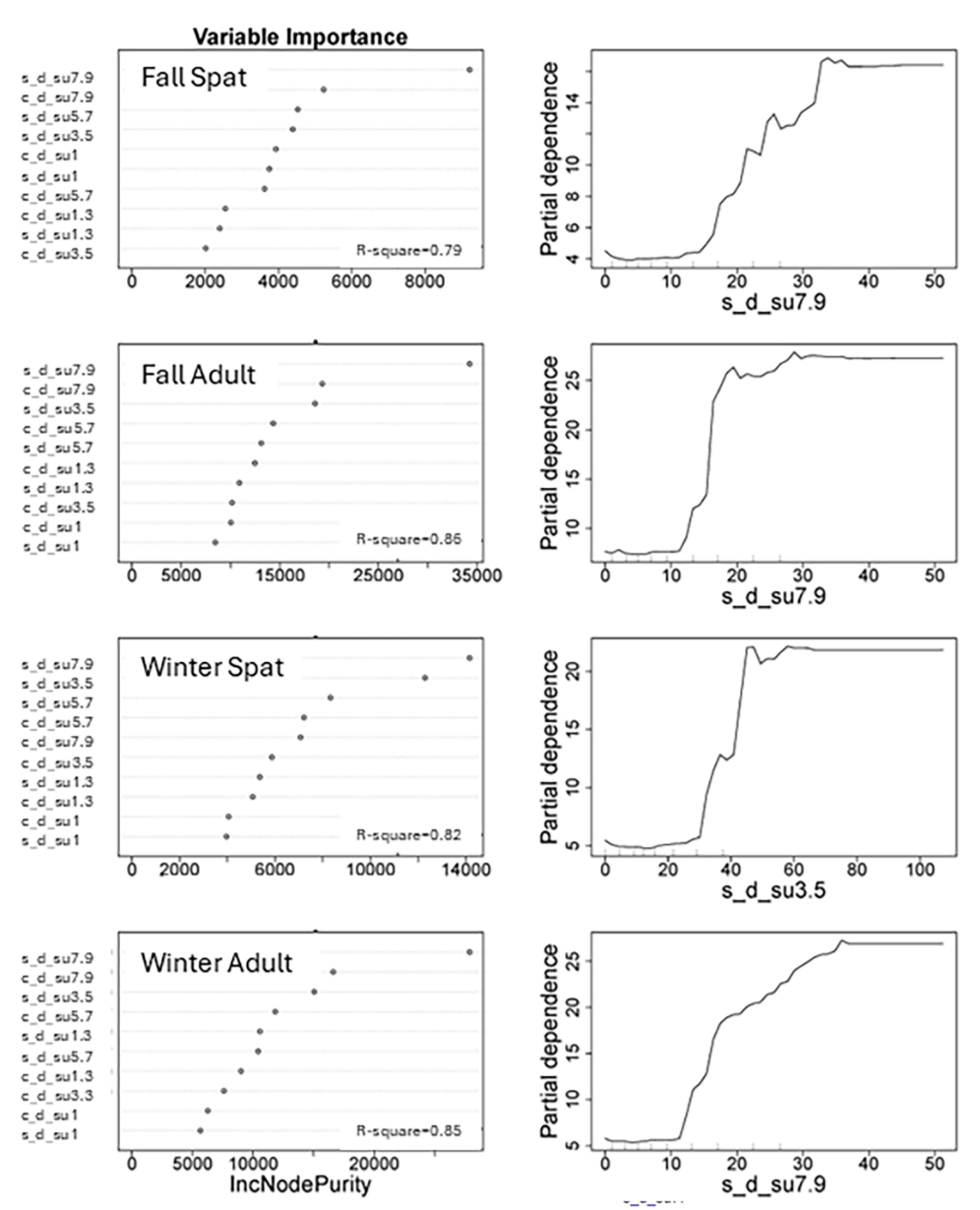
**

**Supplementary Figure S4.** Variable importance and partial dependence plot of variables identified for interpretation as critical lower salinity threshold spat (< 25 mm shell height) or adult (≥ 25 mm shell height) abundance during fall (September/October) or winter (November/December) monitoring completed by Louisiana Department of Wildlife and Fisheries during their long-term oyster monitoring collection for Louisiana West Basins (Atchafalaya + Vermilion + Calcasieu basins). Variable used for importance was either the variable of highest importance, or, when the top three variables were clustered in importance, the lowest salinity range of the top three variables. Variables explored included the total number (s) or the maximum continuous number (c) of days where salinity was within summer (su) salinity bins of < 1, 1 - <3, 3 < 5, 5 < 7, 7 < 9, and are coded such that the variable for total number of days of salinity between within the 7 < 9 bin is s_d_su7.9, or maximum continuous number of days within the 7 < 9 bin is c.d_su7.9.

**
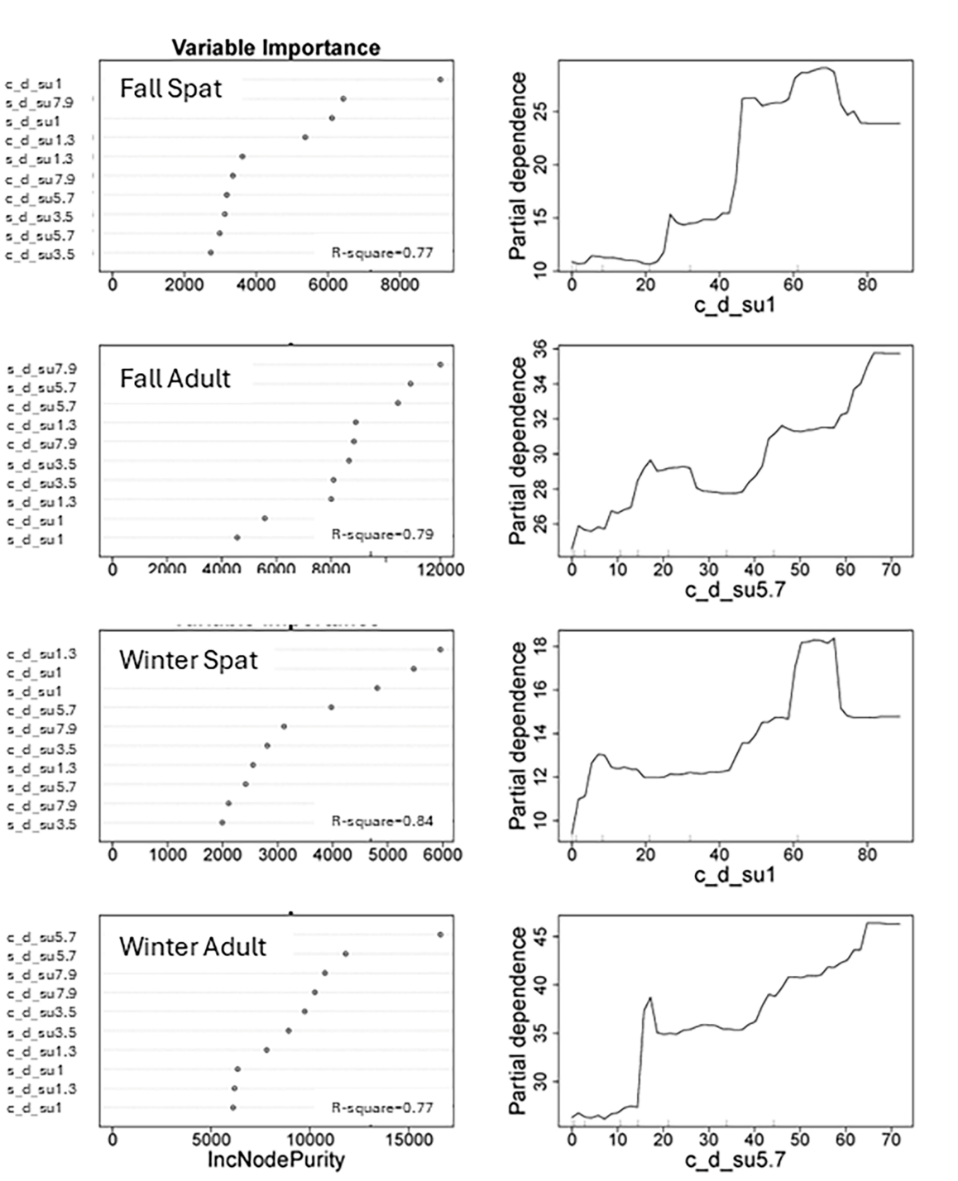
**

**Supplementary Code 1**

1. **Random Forest code**

library("XLConnect")

library(randomForest)

r2 <- function(y,pred){

#Calculate R2

r2 <- 1-mean((y-pred)^2)/mean((y-mean(y))^2)

#Calculate RMSE

rmse <- sqrt(mean((y-pred)^2))

out <- c(round(r2,d=2),round(rmse,d=4))

out

}

fname="Low_salinity_oyster_June_22_2023-for-models-subsets-rev.xlsx"

varImpPlot2=function (x, sort = TRUE, n.var = min(30, nrow(x$importance)),

type = NULL, class = NULL, scale = TRUE, main = deparse(substitute(x)),

ylab=NULL,...)

{

if (!inherits(x, "randomForest"))

stop("This function only works for objects of class `randomForest'")

imp <- importance(x, class = class, scale = scale, type = type,

...)

if (ncol(imp) > 2)

imp <- imp[, -(1:(ncol(imp) - 2))]

nmeas <- ncol(imp)

if (nmeas > 1) {

op <- par(mfrow = c(1, 2), mar = c(4, 6, 4, 1), mgp = c(2,

1, 0), oma = c(0, 0, 2, 0), no.readonly = TRUE)

on.exit(par(op))

}

for (i in 1:nmeas) {

ord <- if (sort)

rev(order(imp[, i], decreasing = TRUE)[1:n.var])

else 1:n.var

xmin <- if (colnames(imp)[i] %in% c("IncNodePurity",

"MeanDecreaseGini"))

0

else min(imp[ord, i])

dotchart(imp[ord, i], xlab = colnames(imp)[i], ylab = ylab,

main = if (nmeas == 1)

main

else NULL, xlim = c(xmin, max(imp[, i])), ...)

}

if (nmeas > 1)

mtext(outer = TRUE, side = 3, text = main, cex = 1.2)

invisible(imp)

}

####### east-SepOct #######

tmp=readWorksheetFromFile(fname,sheet=4)

n=nrow(tmp)

y=as.matrix(tmp[1:n,6:7])

mode(y)="numeric"

yname=c("spat_SepOct","adult_SepOct")

x=as.matrix(tmp[1:n,8:17])

mode(x)="numeric"

xname=colnames(x)

pdf("fig2-east-SepOct-1-21-24.pdf", height=9, width=14.5)

par(mfrow=c(2,2), mar=c(4.5,8,2.5,2), cex.lab=2.5, cex.axis=2, cex.main=2.5)

for (j in 1:2){

indx=which(!is.na(y[,j]))

dat=as.data.frame(x[indx,])

dat$y=y[indx,j]

set.seed(1)

fit=randomForest(y~.,data=dat,importance=T)

prd=predict(fit,dat)

rsq=r2(dat$y,prd)[1]

ylab="East: Fall Spat"

if (j==2){

ylab="East: Fall Adult"

}

varImpPlot2(fit,type=2,main="Variable Importance",ylab=ylab)

legend("bottomright",legend=paste("R-square=",rsq))

vname=xname[order(importance(fit,type=2),decreasing=T)[1]]

partialPlot(fit,dat,vname[1],main="",xlab=vname[1],ylab="Partial dependence")

}

dev.off()

####### east-NovDec #######

tmp=readWorksheetFromFile(fname,sheet=3)

n=nrow(tmp)

y=as.matrix(tmp[1:n,6:7])

mode(y)="numeric"

yname=colnames(y)

#summer salinity variables only

x=as.matrix(tmp[1:n,8:27])[,c(3:4,7:8,11:12,15:16,19:20)]

mode(x)="numeric"

xname=colnames(x)

pdf("fig2-east-NovDec-1-21-24.pdf", height=9, width=14.5)

par(mfrow=c(2,2), mar=c(4.5,8,2.5,2), cex.lab=2.5, cex.axis=2, cex.main=2.5)

for (j in 1:2){

indx=which(!is.na(y[,j]))

dat=as.data.frame(x[indx,])

dat$y=y[indx,j]

set.seed(1)

fit=randomForest(y~.,data=dat,importance=T)

prd=predict(fit,dat)

rsq=r2(dat$y,prd)[1]

ylab="East: Winter Spat"

if (j==2){

ylab="East: Winter Adult"

}

varImpPlot2(fit,type=2,main="Variable Importance",ylab=ylab)

legend("bottomright",legend=paste("R-square=",rsq))

vname=xname[order(importance(fit,type=2),decreasing=T)[1:3]]

if (j==1){partialPlot(fit,dat,vname[2],main="",xlab=vname[2],ylab="Partial dependence")}

if (j==2){partialPlot(fit,dat,vname[1],main="",xlab=vname[1],ylab="Partial dependence")}

}

dev.off()

####### west-SepOct #######

tmp=readWorksheetFromFile(fname,sheet=7)

n=nrow(tmp)

y=as.matrix(tmp[1:n,6:7])

mode(y)="numeric"

yname=c("spat_SepOct","adult_SepOct")

x=as.matrix(tmp[1:n,8:17])

mode(x)="numeric"

xname=colnames(x)

pdf("fig2-west-SepOct-1-21-24.pdf", height=9, width=14.5)

par(mfrow=c(2,2), mar=c(4.5,8,2.5,2), cex.lab=2.5, cex.axis=2, cex.main=2.5)

for (j in 1:2){

indx=which(!is.na(y[,j]))

dat=as.data.frame(x[indx,])

dat$y=y[indx,j]

set.seed(1)

fit=randomForest(y~.,data=dat,importance=T)

prd=predict(fit,dat)

rsq=r2(dat$y,prd)[1]

ylab="West: Fall Spat"

if (j==2){

ylab="West: Fall Adult"

}

varImpPlot2(fit,type=2,main="Variable Importance",ylab=ylab)

legend("bottomright",legend=paste("R-square=",rsq))

vname=xname[order(importance(fit,type=2),decreasing=T)[1:3]]

if (j==1){partialPlot(fit,dat,vname[1],main="",xlab=vname[1],ylab="Partial dependence")}

if (j==2){partialPlot(fit,dat,vname[3],main="",xlab=vname[3],ylab="Partial dependence")}

}

dev.off()

####### west-NovDec #######

tmp=readWorksheetFromFile(fname,sheet=6)

n=nrow(tmp)

y=as.matrix(tmp[1:n,6:7])

mode(y)="numeric"

yname=colnames(y)

#summer salinity variables only

x=as.matrix(tmp[1:n,8:27])[,c(3:4,7:8,11:12,15:16,19:20)]

mode(x)="numeric"

xname=colnames(x)

pdf("fig2-west-NovDec-1-21-24.pdf", height=9, width=14.5)

par(mfrow=c(2,2), mar=c(4.5,8,2.5,2), cex.lab=2.5, cex.axis=2, cex.main=2.5)

for (j in 1:2){

indx=which(!is.na(y[,j]))

dat=as.data.frame(x[indx,])

dat$y=y[indx,j]

set.seed(1)

fit=randomForest(y~.,data=dat,importance=T)

prd=predict(fit,dat)

rsq=r2(dat$y,prd)[1]

ylab="East: Winter Spat"

if (j==2){

ylab="East: Winter Adult"

}

varImpPlot2(fit,type=2,main="Variable Importance",ylab=ylab)

legend("bottomright",legend=paste("R-square=",rsq))

vname=xname[order(importance(fit,type=2),decreasing=T)[1:3]]

if (j==2){partialPlot(fit,dat,vname[1],main="",xlab=vname[1],ylab="Partial dependence")}

if (j==1){partialPlot(fit,dat,vname[2],main="",xlab=vname[2],ylab="Partial dependence")}

}

dev.off()

**Supplementary Code 2**

1. **JAGS code for the model in Figure 2**

#This JAGS (Just Another Gibbs Sampler) code corresponds to the conceptual model (Figure 2)

# Anything after # is comments instead of executable codes

model {

gamma1 ~ dnorm(0, 0.00001) # Eq 6

gamma2 ~ dnorm(0, 0.00001) # Eq 7

gamma3 ~ dnorm(0, 0.00001) # Eq 8

gamma4 ~ dnorm(0, 0.00001) # Eq 9

gamma5 ~ dnorm(0, 0.00001) # Eq 10

taui ~ dgamma(0.01, 0.01) # Eq 12

tauj ~ dgamma(0.01, 0.01) # Eq 13

tauk ~ dgamma(0.01, 0.01) # Eq 14

tau ~ dgamma(0.01, 0.01) # Eq 11

for (i in 1:3) {

bBasin[i] ~ dnorm(0, taui) # - Eq 4 (basin scale)

for (j in 1:87) {

bStation[i,j] ~ dnorm(bBasin[i], tauj) # - Eq 3 (station scale)

}

}

for (i in 1:5) {

bYear[i] ~ dnorm(0, tauk) # - Eq 5 (Year)

}

for (i in 1:441) {

# Calculating mean of spat abundance in fall (Eq 2)

spat_sepoct.mu[i] <- bStation[Mbasin[i],Mstation[i]]+bYear[Myear[i]]+a1*c_d_su1[i]+a2*c_d_su1.3[i]+a3*c_d_su3.5[i]+a4*c_d_su5.7[i]+a5*c_d_su7.9[i]

# Likelihood (Eq 1) spat_sepoct is the data

spat_sepoct[i] ~ dnorm(spat_sepoct.mu[i], tau)

}

}
